# Single nucleotide polymorphisms in DKK3 gene are associated with prostate cancer risk and progression

**DOI:** 10.1590/S1677-5538.IBJU.2014.0041

**Published:** 2015

**Authors:** Min Su Kim, Ha Na Lee, Hae Jong Kim, Soon Chul Myung

**Affiliations:** 1 Department of Urology, Seoul Medical Center, Seoul, Korea; 2 Department of Urology, Seoul Seonam Hospital, EwhaWomans University, Seoul, Korea; 3 Research Institue for Biomedical and Pharmaceutical Sciences, Chung-Ang University, Seoul, Korea; 4 Advanced Urogenital Diseas Research Center, Chung-Ang University, College of Medicine, Seoul, Korea; 5 Department of Urology, Chung-Ang University, College of Medicine, Seoul, Korea

**Keywords:** Biological Markers, Genetic Variation, Prostatic Neoplasms, Polymorphism, Single Nucleotide

## Abstract

We had investigated whether sequence variants within DKK3 gene are associated with the development of prostate cancer in a Korean study cohort. We evaluated the association between 53 single nucleotide polymorphisms (SNPs) in the DKK3 gene and prostate cancer risk as well as clinical characteristics (PSA, clinical stage, pathological stage and Gleason score) in Korean men (272 prostate cancer subjects and 173 benign prostate hyperplasia subjects) using unconditional logistic regression analysis. Of the 53 SNPs and 25 common haplotypes, 5 SNPs and 4 haplotypes were associated with prostate cancer risk (P=0.02–0.04); 3 SNPs and 2 haplotypes were significantly associated with susceptibility to prostate cancer, however 2 SNPs and 2 haplotypes exhibited a significant protective effect on prostate cancer. Logistic analyses of the DKK3 gene polymorphisms with several prostate cancer related factors showed that several SNPs were significant; three SNPs and two haplotypes to PSA level, three SNPs and two haplotypes to clinical stage, nine SNPs and two haplotype to pathological stage, one SNP and one haplotypes to Gleason score. To the author's knowledge, this is the first report documenting that DKK3 polymorphisms are not only associated with prostate cancer but also related to prostate cancer-related factors.

## Introduction

Prostate cancer is one of the most common cancers in men. Rates of detection of prostate cancer vary widely across the world, with less frequent detection in South and East Asia than in Europe and especially the United States (1, 2). However the incidence rate of prostate cancer in Korea has rised rapidly during the last decade (3). The etiology of prostate cancer is largely unknown, although several risk factors such as diet, occupation, sexually transmitted agents were investigated epidemiologically; the only established risk factors for prostate cancer are increased age, ethnic background and familial history (4). Recently genome-wide association studies (GAWS) have identified more than 40 single-nucleotide polymorphisms (SNPs) on various genes or chromosomal loci that are significantly associated with prostate cancer susceptibility (5). There is increasing interest in investigating the potential usefulness of SNPs as diagnostic and prognostic biomarkers for prostate cancer outcomes (6, 7).

The wingless-type mouse mammary tumor virus integration site (Wnt) signaling pathway describes a complex network of proteins well known for their roles in embryogenesis and tumorigenesis (8). Uncontrolled Wnt signaling has been recognized as an important trait of human cancer (9). Their activity is regulated by the secreted Wnt signaling inhibitors including Wnt antagonist families namely the secreted frizzled-related protein (sFRP), Wnt inhibitory factor 1 (Wif-1), and Dickkopf (DKK1–4) families (10).

Dickkopf homologue 3 (DKK3) gene which is located at 11p15, is proposed to function as a tumor suppressor gene since its expression is down-regulated in many types of cancer cells (11). Inactivation of tumor-suppressive genes by either genetic or epigenetic mechanisms contributes to cancer formation. Ectopic expression of DKK3 results in decreased proliferation and is accompanied by attenuation of the mitogen-activated protein kinase pathway (12). If DKK3 regulates the growth of normal and cancerous prostate cells, the variation in DKK3 activity may be important in the onset and progression of prostate cancer. We hypothesized that sequence variations in DKK3 are candidates for risk factors for development of prostate cancer and progression.

However to our knowledge, there have been no reports regarding DKK3 gene polymorphisms in prostate cancer. Here we investigated whether SNPs of the DKK3 gene were associated with the development of prostate cancer in a Korean cohort.

## MATERIALS AND METHODS

### Study Population

Blood samples were obtained from the Korean Prostate Bank (Seoul, Korea). Both prostate cancer and benign prostatic hyperplasia (BPH) groups originated from a population of older men treated at St. Mary's Hospital (Seoul, Korea). Peripheral blood leukocyte samples for genotyping were obtained from 445 men (prostate cancer, n=272; BPH, n=173) and were stored at −80°C. BPH subjects had true biopsy for confirmation for free of prostate cancer at the time when the samples were taken according to prostate-specific antigen blood tests and digital rectal prostate exams and were excluded from the study if they had a history of prostate cancer. Prostate cancer subjects with primary, incident, histologically confirmed prostate cancer were recruited within 6 months of diagnosis. The median age of the BPH cohort was 67.3 years, and the median age of the prostate cancer cohort was 68.2 years. BPH samples were used as the control group for several reasons. First, most men have evidence of BPH by the age of 70 or 80 years; thus, the presence of some degree of BPH is “normal” at the median age of diagnosis in our prostate cancer cohort (age 67.3 years). Truly “normal” samples would thus only be obtained in a much younger control cohort, which could introduce bias. Second, the collection of blood samples requires a hospital visit and a prostate cancer screening procedure, which would only be undertaken in men with evidence of symptoms of prostate enlargement. All the study participants provided written informed consent. The institutional review board of Chung-Ang University Hospital and Catholic University Hospital approved the study. Blood samples were collected in tubes containing sodium ethylene diaminetetraacetic acid from St. Mary's Hospital in Korea. The QIA amp blood extraction kit (Qiagen, Seoul, Korea) was used for DNA extraction.

The PSA level was classified as low (PSA<4), intermediate (4≤PSA<10), or high (PSA≥10). The Gleason score was designated as low (Gleason score 2–6), intermediate (Gleason score 4+3, 3+4), or high (Gleason score 8–10) grade. The clinicopathologic regional stages were categorized as localized (Stage T1N0M0 orT2N0M0), locally advanced (Stage T3N0M0 or T4N0M0), and metastatic (TxN+ or TxM+) according to the pathologic and/or radiologic reports. Clinical characteristics of the study population are listed in Table-1 and were similar to those of a previous Korean study (13).

**Table 1:** study characteristics of prostate cancer cases and controls.

		Cases	Controls
N		272	173
Age (year)±SD		68.2±6.8	67.3±8.8
BMI in kg/m^2^ (%)		24.1±3.3	24.0±3.0
Prostate volume (cm3)±SD		37.2±18.6	48.4+26.2
PSA, ng/mL (mean±SD)		48.2±192.8	5.2±6.7
**Gleason score, n (%)**			
	low grade	29 (11%)	
	(3+4, 4+3)	202 (75%)	
	high grade	39 (14%)	
**Clinical Stage, n (%)**			
	localized	252 (55.1%)	
	locally advanced	10 (35.7%)	
	metastatic	8 (8.5%)	
	unknown	2 (0.7%)	
**Pathologic Stage, n (%)**			
	Localized (T2)	152 (60.3%)	
	Advanced (≥T3)	100 (39.7%)	

### SNP Selection and Genotyping

We selected 53 SNPs from two international databases (International HapMap and National Center for Biotechnology Information dbSNPs). SNP selection from the International HapMap database (Han Chinese and Japanese) was performed as follows: (a) extraction of all genotypes from the CHB and JPN population in DKK3 gene region using HapMart of the International HapMap database (version release 27; available from: http://www.hapmap.org) (b) calculation of minor allele frequency and linkage disequilibrium using Haplo view software (Cambridge, MA; available from: http://www.broad.mit.edu/mpg/haploview), and (c) selection of SNPs having minor allele frequency >0.05 and tagging SNPs if several SNPs showed high linkage disequilibrium >0.98. Furthermore, we added the SNPs in the DKK3 gene region from the National Center for Biotechnology Information db-SNPs. The selection criteria included location (SNPs in exons were preferred) and amino acid changes (nonsynonymous SNPs were preferred). Genotyping was performed at the multiplex level using the Illumina Golden Gate genotyping system (14). In brief, approximately 250 ng genomic DNA extracted from the blood of each subject was used for genotyping by DNA activation, binding to paramagnetic particles, hybridization to oligonucleotides, washing, extension, ligation, amplification by polymerase chain reaction, and hybridization to the Bead plate in an appropriate hybridization buffer. The image intensities were scanned using the Bead Xpress Reader, and genotyped using the Genome Studio software (Illumina). The genotype quality score for retaining data was set to 0.25. A total of 53 SNPs were successfully genotyped.

### Statistical analysis

The SNP genotype frequencies were examined for Hardy-Weinberg equilibrium using the chi-square test, and all were found to be consistent (P>0.05) with Hardy-Weinberg equilibrium among the Korean controls. The data were analyzed using unconditional logistic regression analysis to calculate the odds ratio (OR) as an estimate of the relative risk of prostate cancer associated with SNP genotypes (15).

To determine the association between the genotype and haplotype distributions of patients and controls, logistic analysis was performed, controlling for age (continuous value) as a covariate to eliminate or reduce any confounding influence. Significant associations were indicated (P≤0.05). Multiple comparisons were also accounted for by using per mutations to calculate the exact P values for each significant SNP (α=0.05). Lewontin's D' and the linkage disequilibrium co-efficient r2 were examined to measure linkage disequilibrium between all pairs of bi-allelic loci (16). Haplotypes were inferred from the successfully genotyped SNPs using the PHASE algorithm, version 2.0 (17), and association analysis was performed using SAS, version 9.1 (SAS Institute, Cary, NC). To achieve optimal correction for multiple testing of markers, representing SNPs in linkage disequilibrium with each other, the effective number of independent marker loci (21.3) was calculated using SNP spectral decomposition software (available from: http://genepi.qimr.edu.au/general/daleN/SNPSpD/), a program that is based on the spectral decomposition of matrices of pair-wise linkage disequilibrium among markers (18).

Statistical power of single associations was calculated with false positive rate of 5%, disease lifetime prevalence of 0.02%, given minor allele frequencies and sample sizes, and assuming a relative risk of 1.5, using PGA (Power for Genetic Association Analyses) software (19).

## RESULTS

### The association between DKK3 polymorphisms and the risk of prostate cancer

**Figure 1:**
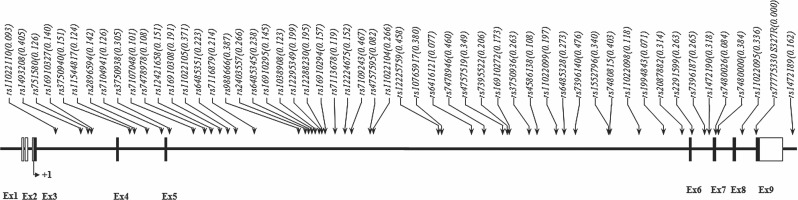
Map of DKK3 (dickkopf 3 homolog) on chromosome 11p15.2 (46.38 kb).

**Figure 2:**
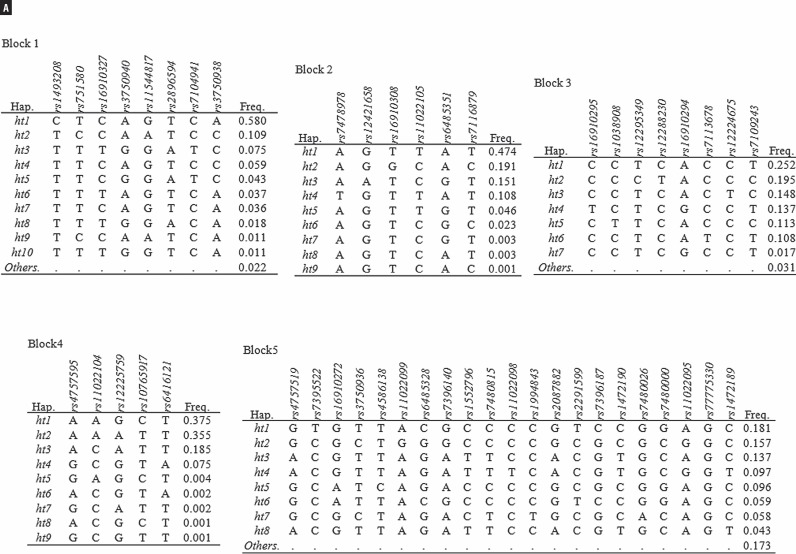

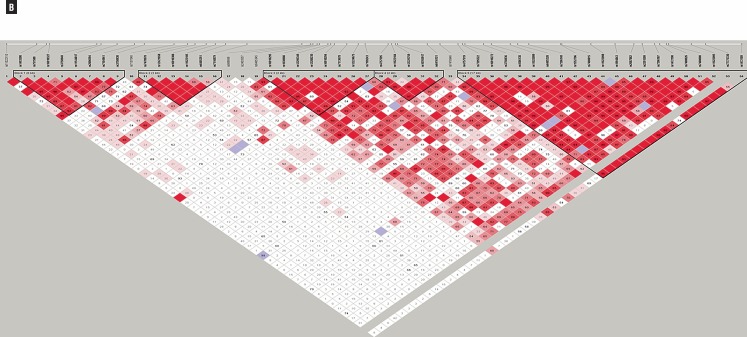
Haplotypes among DKK3 SNPs. A) Haplotypes in DKK3. “Others” category contains rare haplotypes, B) Linkage disequilibrium among DKK3 polymorphisms.

A total of 53 SNPs from the human DKK3 gene in 272 patients with prostate cancer and 173 control subjects were successfully genotyped to determine the potential association of the gene with the development of prostate cancer (Figure-1). The genotype distributions in the control group were in Hardy-Weinberg equilibrium (P>0.05; data not shown). The measured linkage disequilibrium among 53 SNPs was determined by calculating Lewontin's D' and r^2^ values; the results showed that these SNPs were divided among five haplotype blocks (Figure-2). The allele frequencies of each of the polymorphisms and common haplotypes were compared between the patients and the normal controls using logistic regression models. The results of the analysis revealed that five SNPs showed nominal evidence of an association at a P<0.05 level of significance (Table-2, Supplementary Table-1). Of the five significantly associated SNPs, three (rs12421658, rs11022105, and rs4586138) showed a greater frequency in patients with prostate cancer than in the normal controls (OR 1.63, p=0.04; OR 1.54, p=0.04; and OR 1.89, p=0.02, respectively). In addition, a haplotype association test was performed on 25 common haplotypes (frequency >0.05) within the five haplotype blocks. Two haplotypes (Block2_ht3 and Block5_ht5) showed a marginal association with the risk of prostate cancer (P=0.04 and P=0.03, respectively). There were two SNPs (rs2087882 and rs1472190) and two haplotypes (Block3_ht6 and Block5_ht4) that exhibited a significant protective effect from prostate cancer (Table-2).

**Table 2:** Logistic regression analysis of DKK3 SNPs with the risk of prostate cancer in Korean Population.

SNPID	Chr	Position	Alleles	Minor Allele Frequency		Co-dominant		Dominant		Recessive	
				Pca (n=272)	BPH (n=173)	OR(95%CI)	P-value	OR(95%CI)	P-value	OR(95%CI)	P-value
rs12421658	11	Intron	G>A	0.167	0.113	1.59(1.05–2.41)	**0.03** *	1.63(1.03–2.59)	**0.04** *	2.44(0.50–11.93)	0.27
rs11022105	11	Intron	T>C	0.395	0.318	1.46(1.08–1.99)	**0.02** *	1.54(1.03–2.30)	**0.04** *	1.83(0.95–3.52)	0.07
rs4586138	11	Intron	T>C	0.136	0.078	1.75(1.09–2.80)	**0.02** *	1.89(1.12–3.17)	**0.02** *	1.85(0.34–9.97)	0.47
rs2087882	11	Intron	G>A	0.287	0.358	0.73(0.55–0.97)	**0.03** *	0.67(0.45–0.99)	**0.04** *	0.64(0.36–1.17)	0.15
rs1472190	11	Intron	C>T	0.290	0.361	0.74(0.56–0.98)	**0.04** *	0.69(0.47–1.03)	**0.07**	0.61(0.34–1.10)	0.10
DKK3_B2_ht3	11	․	․	0.167	0.113	1.59(1.05–2.41)	**0.03** *	1.63(1.03–2.59)	**0.04** *	2.44(0.50–11.93)	0.27
DKK3_B3_ht6	11	․	․	0.092	0.142	0.62(0.40–0.96)	**0.03** *	0.58(0.36–0.94)	**0.03** *	0.65(0.12–3.47)	0.62
DKK3_B5_ht4	11	․	․	0.077	0.127	0.55(0.34–0.88)	**0.01** *	0.57(0.35–0.95)	**0.03** *	․	․
DKK3_B5_ht5	11	․	․	0.119	0.069	1.67(1.02–2.73)	**0.04** *	1.80(1.04–3.12)	**0.03** *	1.85(0.34–9.97)	0.47

Minor allele frequencies and P-values for logistic analyses of three alternative models (co-dominant, dominant and recessive models) controlling for age as covariate are shown. OR: odd ratio, CI: confidence interval

* Statistically significant at P ≤ 0.05

**Supplementary Table 1:** Logistic regression analysis of DKK3 SNPs with the risk of prostate cancer in Korean Population.

SNPID	Chr	Position	AA Change	Alleles	Minor Allele Frequency		Co-dominant		Dominant		Recessive	
					Pca (n=272)	BPH (n=173)	OR(95%CI)	P-value	OR(95%CI)	P-value	OR(95%CI)	P-value
rs11022110	11	Intron	․	G>A	0.097	0.081	1.26(0.76–2.09)	0.36	1.21(0.72–2.04)	0.48	․	․
rs1493208	11	Intron	․	C>T	0.412	0.395	1.13(0.84–1.54)	0.42	1.31(0.86–2.00)	0.21	0.95(0.54–1.68)	0.86
rs751580	11	Intron	․	T>C	0.134	0.113	1.34(0.86–2.08)	0.19	1.26(0.79–2.00)	0.34	․	․
rs16910327	11	Intron	․	C>T	0.136	0.156	0.82(0.55–1.22)	0.32	0.85(0.55–1.32)	0.47	0.38(0.08–1.76)	0.21
rs3750940	11	Intron	․	A>G	0.145	0.162	0.89(0.60–1.32)	0.55	0.90(0.58–1.38)	0.62	0.63(0.13–2.98)	0.56
rs11544817	11	Intron	․	G>A	0.131	0.113	1.30(0.84–2.01)	0.24	1.20(0.75–1.92)	0.45	․	․
rs2896594	11	Intron	․	T>A	0.140	0.147	0.94(0.63–1.41)	0.76	0.96(0.62–1.49)	0.86	0.65(0.14–3.05)	0.58
rs7104941	11	Intron	․	C>T	0.126	0.124	1.04(0.68–1.58)	0.87	1.08(0.68–1.72)	0.76	0.71(0.15–3.32)	0.66
rs3750938	11	Intron	․	A>C	0.315	0.283	1.29(0.93–1.77)	0.13	1.25(0.84–1.87)	0.27	1.87(0.84–4.19)	0.13
rs7107048	11	Intron	․	C>T	0.101	0.099	1.04(0.67–1.61)	0.88	1.02(0.61–1.69)	0.96	1.29(0.30–5.51)	0.73
rs7478978	11	Intron	․	A>T	0.110	0.107	1.02(0.65–1.61)	0.94	1.02(0.63–1.65)	0.95	1.09(0.11–10.79)	0.94
rs12421658	11	Intron	․	G>A	0.167	0.113	1.59(1.05–2.41)	**0.03**	1.63(1.03–2.59)	**0.04**	2.44(0.50–11.93)	0.27
rs16910308	11	Intron	․	T>G	0.205	0.165	1.29(0.89–1.87)	0.18	1.39(0.91–2.11)	0.13	1.02(0.33–3.20)	0.97
rs11022105	11	Intron	․	T>C	0.395	0.318	1.46(1.08–1.99)	**0.02**	1.54(1.03–2.30)	**0.04**	1.83(0.95–3.52)	0.07
rs6485351	11	Intron	․	A>G	0.222	0.211	1.06(0.76–1.47)	0.74	1.10(0.73–1.65)	0.66	0.98(0.42–2.29)	0.96
rs7116879	11	Intron	․	T>C	0.220	0.202	1.15(0.81–1.63)	0.44	1.23(0.82–1.84)	0.32	0.88(0.32–2.44)	0.81
rs988666	11	Intron	․	C>T	0.401	0.387	1.03(0.77–1.39)	0.83	0.90(0.59–1.35)	0.60	1.41(0.78–2.53)	0.25
rs2403557	11	Intron	․	C>T	0.265	0.257	1.06(0.77–1.44)	0.74	1.07(0.72–1.59)	0.75	1.08(0.52–2.28)	0.83
rs6485345	11	Intron	․	G>A	0.255	0.214	1.28(0.92–1.79)	0.15	1.39(0.93–2.07)	0.11	1.19(0.50–2.83)	0.69
rs16910295	11	Intron	․	C>T	0.144	0.145	0.99(0.66–1.49)	0.97	0.97(0.62–1.51)	0.89	1.41(0.24–8.24)	0.70
rs1038908	11	Intron	․	C>T	0.131	0.107	1.30(0.83–2.06)	0.26	1.42(0.89–2.29)	0.14	․	․
rs12295349	11	Intron	․	T>C	0.182	0.211	0.82(0.58–1.16)	0.26	0.88(0.58–1.33)	0.54	0.41(0.15–1.14)	0.09
rs12288230	11	Intron	․	C>T	0.178	0.205	0.82(0.58–1.17)	0.28	0.89(0.59–1.34)	0.57	0.41(0.15–1.14)	0.09
rs16910294	11	Intron	․	A>G	0.164	0.145	1.14(0.77–1.70)	0.51	1.06(0.69–1.64)	0.79	4.73(0.57–39.47)	0.15
rs7113678	11	Intron	․	C>T	0.105	0.147	0.68(0.44–1.04)	0.07	0.65(0.41–1.03)	0.07	0.65(0.12–3.47)	0.62
rs12224675	11	Intron	․	C>T	0.164	0.142	1.20(0.82–1.76)	0.36	1.24(0.80–1.94)	0.33	1.18(0.36–3.90)	0.78
rs7109243	11	Intron	․	T>C	0.469	0.457	1.04(0.79–1.38)	0.77	1.06(0.69–1.63)	0.80	1.06(0.66–1.71)	0.81
rs4757595	11	Intron	․	A>G	0.086	0.078	1.07(0.64–1.81)	0.79	1.12(0.66–1.91)	0.68	․	․
rs11022104	11	Intron	․	A>C	0.254	0.266	0.93(0.68–1.28)	0.65	0.95(0.64–1.41)	0.79	0.80(0.37–1.72)	0.56
rs12225759	11	Intron	․	A>G	0.461	0.465	0.97(0.73–1.29)	0.82	1.22(0.79–1.89)	0.38	0.71(0.44–1.16)	0.17
rs10765917	11	Intron	․	T>C	0.378	0.393	0.93(0.69–1.25)	0.63	0.94(0.62–1.41)	0.75	0.87(0.49–1.53)	0.62
rs6416121	11	Intron	․	T>A	0.083	0.069	1.15(0.67–1.98)	0.61	1.21(0.69–2.12)	0.50	․	․
rs7478946	11	Intron	․	T>C	0.454	0.460	0.99(0.75–1.32)	0.97	1.23(0.80–1.90)	0.35	0.75(0.46–1.23)	0.25
rs4757519	11	Intron	․	G>A	0.331	0.384	0.80(0.61–1.06)	0.12	0.74(0.49–1.10)	0.14	0.76(0.44–1.30)	0.31
rs7395522	11	Intron	․	C>T	0.193	0.211	0.92(0.66–1.29)	0.63	0.91(0.60–1.37)	0.65	0.89(0.35–2.21)	0.79
rs16910272	11	Intron	․	G>A	0.189	0.159	1.18(0.83–1.69)	0.35	1.28(0.83–1.97)	0.27	1.04(0.40–2.70)	0.93
rs3750936	11	Intron	․	T>C	0.279	0.240	1.24(0.89–1.72)	0.20	1.35(0.91–2.01)	0.13	1.05(0.45–2.43)	0.91
rs4586138	11	Intron	․	T>C	0.136	0.078	1.75(1.09–2.80)	**0.02**	1.89(1.12–3.17)	**0.02**	1.85(0.34–9.97)	0.47
rs11022099	11	Intron	․	A>G	0.211	0.174	1.30(0.90–1.86)	0.16	1.48(0.97–2.25)	0.07	0.76(0.27–2.13)	0.60
rs6485328	11	Intron	․	G>C	0.248	0.295	0.82(0.60–1.11)	0.19	0.75(0.50–1.11)	0.15	0.85(0.42–1.74)	0.66
rs7396140	11	Intron	․	A>G	0.461	0.477	0.98(0.75–1.27)	0.86	0.91(0.59–1.40)	0.67	1.03(0.66–1.63)	0.89
rs1552796	11	Intron	․	C>T	0.322	0.373	0.81(0.61–1.07)	0.14	0.76(0.51–1.13)	0.17	0.74(0.42–1.30)	0.30
rs7480815	11	Intron	․	C>T	0.389	0.434	0.83(0.63–1.09)	0.18	0.78(0.52–1.18)	0.25	0.77(0.47–1.26)	0.30
rs11022098	11	Intron	․	C>T	0.104	0.142	0.68(0.43–1.05)	0.08	0.73(0.46–1.18)	0.20	․	․
rs1994843	11	Intron	․	C>T	0.072	0.072	0.94(0.54–1.62)	0.81	0.98(0.56–1.71)	0.93	․	․
rs2087882	11	Intron	․	G>A	0.287	0.358	0.73(0.55–0.97)	**0.03**	0.67(0.45–0.99)	**0.04**	0.64(0.36–1.17)	0.15
rs2291599	11	Intron	․	C>T	0.237	0.292	0.79(0.58–1.07)	0.13	0.72(0.49–1.08)	0.11	0.79(0.38–1.63)	0.52
rs7396187	11	Intron	․	G>C	0.241	0.292	0.80(0.59–1.09)	0.16	0.72(0.49–1.08)	0.11	0.86(0.42–1.76)	0.69
rs1472190	11	Intron	․	C>T	0.290	0.361	0.74(0.56–0.98)	**0.04**	0.69(0.47–1.03)	0.07	0.61(0.34–1.10)	0.10
rs7480026	11	Intron	․	G>A	0.086	0.081	1.06(0.64–1.77)	0.81	1.07(0.63–1.83)	0.80	0.91(0.06–14.97)	0.95
rs7480000	11	Intron	․	C>G	0.391	0.370	1.09(0.83–1.45)	0.54	1.22(0.81–1.82)	0.34	0.97(0.57–1.68)	0.92
rs11022095	11	Intron	․	A>G	0.339	0.329	1.04(0.78–1.41)	0.77	1.12(0.75–1.66)	0.59	0.92(0.49–1.73)	0.80
rs1472189	11	3'flanking	․	C>T	0.143	0.191	0.71(0.49–1.02)	0.07	0.76(0.50–1.17)	0.21	0.24(0.07–0.82)	0.02
DKK3_B1_ht1	11	․	․	․	0.426	0.413	1.10(0.82–1.49)	0.52	1.16(0.76–1.78)	0.49	1.09(0.62–1.89)	0.77
DKK3_B1_ht2	11	․	․	․	0.114	0.098	1.32(0.83–2.11)	0.25	1.26(0.77–2.05)	0.36	․	․
DKK3_B1_ht3	11	․	․	․	0.077	0.072	1.09(0.64–1.88)	0.75	1.15(0.66–2.02)	0.62	․	․
DKK3_B1_ht4	11	․	․	․	0.063	0.052	1.27(0.69–2.34)	0.45	1.24(0.66–2.32)	0.51	․	․
DKK3_B2_ht1	11	․	․	․	0.458	0.514	0.81(0.61–1.08)	0.15	0.79(0.50–1.25)	0.31	0.73(0.45–1.17)	0.19
DKK3_B2_ht2	11	․	․	․	0.204	0.165	1.28(0.89–1.85)	0.19	1.38(0.90–2.09)	0.14	1.02(0.33–3.18)	0.98
DKK3_B2_ht3	11	․	․	․	0.167	0.113	1.59(1.05–2.41)	**0.03**	1.63(1.03–2.59)	**0.04**	2.44(0.50–11.93)	0.27
DKK3_B2_ht4	11	․	․	․	0.110	0.107	1.02(0.65–1.61)	0.94	1.02(0.63–1.65)	0.95	1.09(0.11–10.79)	0.94
DKK3_B3_ht1	11	․	․	․	0.259	0.243	1.09(0.80–1.48)	0.60	1.12(0.75–1.66)	0.59	1.10(0.54–2.27)	0.79
DKK3_B3_ht2	11	․	․	․	0.178	0.205	0.82(0.58–1.17)	0.28	0.89(0.59–1.34)	0.57	0.41(0.15–1.14)	0.09
DKK3_B3_ht3	11	․	․	․	0.158	0.142	1.15(0.78–1.68)	0.48	1.18(0.75–1.84)	0.47	1.17(0.36–3.87)	0.79
DKK3_B3_ht4	11	․	․	․	0.134	0.139	0.96(0.63–1.47)	0.86	0.91(0.58–1.43)	0.69	2.81(0.29–27.01)	0.37
DKK3_B3_ht5	11	․	․	․	0.118	0.101	1.22(0.76–1.94)	0.41	1.33(0.82–2.16)	0.25	․	․
DKK3_B3_ht6	11	․	․	․	0.092	0.142	0.62(0.40–0.96)	**0.03**	0.58(0.36–0.94)	**0.03**	0.65(0.12–3.47)	0.62
DKK3_B4_ht1	11	․	․	․	0.373	0.387	0.94(0.70–1.25)	0.66	0.97(0.65–1.46)	0.88	0.83(0.47–1.46)	0.51
DKK3_B4_ht2	11	․	․	․	0.369	0.341	1.15(0.86–1.52)	0.35	1.41(0.95–2.10)	0.09	0.85(0.48–1.50)	0.57
DKK3_B4_ht3	11	․	․	․	0.167	0.191	0.86(0.60–1.23)	0.40	0.89(0.58–1.35)	0.58	0.55(0.19–1.59)	0.27
DKK3_B4_ht4	11	․	․	․	0.081	0.066	1.19(0.69–2.05)	0.54	1.25(0.71–2.21)	0.44	․	․
DKK3_B5_ht1	11	․	․	․	0.165	0.188	0.90(0.64–1.29)	0.57	0.89(0.58–1.35)	0.57	0.88(0.33–2.34)	0.79
DKK3_B5_ht2	11	․	․	․	0.162	0.150	1.12(0.76–1.65)	0.56	1.16(0.75–1.79)	0.52	1.03(0.29–3.70)	0.97
DKK3_B5_ht3	11	․	․	․	0.129	0.150	0.83(0.57–1.21)	0.34	0.81(0.52–1.28)	0.37	0.72(0.24–2.16)	0.55
DKK3_B5_ht4	11	․	․	․	0.077	0.127	0.55(0.34–0.88)	**0.01**	0.57(0.35–0.95)	**0.03**	․	․
DKK3_B5_ht5	11	․	․	․	0.119	0.069	1.67(1.02–2.73)	**0.04**	1.80(1.04–3.12)	**0.03**	1.85(0.34–9.97)	0.47
DKK3_B5_ht6	11	․	․	․	0.050	0.075	0.66(0.37–1.17)	0.16	0.64(0.35–1.17)	0.15	0.71(0.04–13.18)	0.82
DKK3_B5_ht7	11	․	․	․	0.063	0.055	1.10(0.59–2.02)	0.77	1.10(0.59–2.02)	0.77	․	․

Minor allele frequencies and P-values for logistic analyses of three alternative models (co-dominant, dominant and recessive models) controlling for age as covariate are shown. Significant associations are shown in boldface (P-value<0.05).

**MAF:** minor allele frequency, OR: odd ratio, CI: confidence interval

### The association between DKK3 polymorphisms and PSA level in prostate cancer group

We performed analyses involving only the patients with prostate cancer. Four SNPs and two haplotypes exhibited a significant association with the PSA levels (Table-3, Supplementary Table-2). Two SNPs (rs16910308, rs7116879) and one haplotype (Block2_ht2) had a markedly significant effect on elevated PSA levels in the co-dominant and dominant model (OR 1.77, p=0.007 and OR 2.27, p=0.0007; OR 1.71, p=0.0009 and OR 2.21, p=0.0008; OR 1.77, p=0.007 and OR 2.27, p=0.0007, respectively). One SNP (rs988666) had a positive association with PSA in the recessive model (OR 1.92, p=0.04), the other SNP (rs16910295) had same result in the co-dominant model (OR 1.64, p=0.04). One haplotype (Block3_ht5) had a negative association with PSA in the co-dominant model (OR 0.59, p=0.05) and dominant model (OR 0.59, p=0.05).

**Table 3:** Logistic analysis of DKK3 polymorphisms according to PSA criteria.

	Minor Allele Frequency			Co-dominant		Dominant		Recessive	
SNPID	PSA>10 (n=113)	4<PSA<10 (n=98)	PSA<4 (n=62)	OR(95%CI)	P-value	OR(95%CI)	P-value	OR(95%CI)	P-value
rs16910308	0.243	0.222	0.105	1.77(1.17–2.67)	**0.007**	2.27(1.41–3.63)	**0.0007**	0.75(0.22–2.55)	0.64
rs7116879	0.257	0.237	0.121	1.71(1.14–2.56)	**0.009**	2.21(1.39–3.52)	**0.0008**	0.78(0.24–2.49)	0.67
rs988666	0.442	0.367	0.379	1.29(0.93–1.78)	0.13	1.17(0.74–1.85)	0.51	1.92(1.02–3.60)	**0.04**
rs16910295	0.173	0.134	0.105	1.64(1.02–2.63)	**0.04**	1.52(0.91–2.52)	0.11	․	․
DKK3_B2_ht2	0.243	0.219	0.105	1.77(1.17–2.67)	**0.007**	2.27(1.42–3.64)	**0.0007**	0.75(0.22–2.55)	0.64
DKK3_B3_ht5	0.080	0.143	0.145	0.59(0.35–0.99)	**0.05**	0.59(0.35–0.99)	**0.05**	․	․

Minor allele frequencies and P-values for logistic analyses of three alternative models (co-dominant, dominant and recessive models) controlling for age as covariate are shown. OR: odd ratio, CI: confidence interval * Statistically significant at P < 0.05

**Supplementary Table 2:** Logistic analysis of DKK3 polymorphisms according to PSA criteria.

	Minor Allele Frequency			Co-dominant		Dominant		Recessive	
SNPID	PSA>10 (n=113)	4<PSA<10 (n=98)	PSA<4 (n=62)	OR(95%CI)	P-value	OR(95%CI)	P-value	OR(95%CI)	P-value
rs11022110	0.097	0.102	0.089	1.05(0.62–1.79)	0.86	1.14(0.64–2.01)	0.65	0.26(0.02–4.04)	0.33
rs1493208	0.394	0.459	0.371	1.01(0.72–1.43)	0.94	1.17(0.73–1.89)	0.51	0.81(0.43–1.52)	0.50
rs751580	0.115	0.158	0.129	0.89(0.56–1.41)	0.62	0.85(0.51–1.41)	0.52	1.27(0.24–6.67)	0.78
rs16910327	0.146	0.143	0.113	1.18(0.73–1.89)	0.50	1.14(0.69–1.89)	0.60	2.71(0.23–31.39)	0.43
rs3750940	0.155	0.168	0.097	1.25(0.79–1.97)	0.35	1.31(0.80–2.15)	0.29	0.98(0.15–6.19)	0.98
rs11544817	0.119	0.143	0.129	0.95(0.60–1.49)	0.81	0.88(0.52–1.47)	0.62	1.70(0.36–8.02)	0.50
rs2896594	0.155	0.158	0.089	1.32(0.83–2.11)	0.24	1.41(0.85–2.33)	0.19	1.03(0.16–6.56)	0.97
rs7104941	0.142	0.141	0.073	1.39(0.85–2.25)	0.19	1.58(0.93–2.70)	0.09	0.74(0.12–4.62)	0.75
rs3750938	0.292	0.361	0.282	0.97(0.69–1.36)	0.85	1.12(0.72–1.75)	0.62	0.62(0.29–1.33)	0.21
rs7107048	0.111	0.102	0.089	1.13(0.69–1.85)	0.63	1.24(0.70–2.20)	0.47	0.76(0.17–3.42)	0.72
rs7478978	0.124	0.102	0.097	1.25(0.75–2.07)	0.40	1.36(0.78–2.35)	0.28	0.41(0.05–3.44)	0.41
rs12421658	0.137	0.168	0.218	0.70(0.46–1.06)	0.10	0.75(0.46–1.21)	0.24	0.27(0.07–1.02)	**0.05**
rs16910308	0.243	0.222	0.105	1.77(1.17–2.67)	**0.007**	2.27(1.41–3.63)	**0.0007**	0.75(0.22–2.55)	0.64
rs11022105	0.398	0.418	0.347	1.16(0.83–1.63)	0.38	1.23(0.77–1.96)	0.38	1.19(0.62–2.27)	0.61
rs6485351	0.186	0.230	0.274	0.73(0.51–1.06)	0.10	0.75(0.47–1.18)	0.21	0.45(0.17–1.19)	0.11
rs7116879	0.257	0.237	0.121	1.71(1.14–2.56)	0.009	2.21(1.39–3.52)	**0.0008**	0.78(0.24–2.49)	0.67
rs988666	0.442	0.367	0.379	1.29(0.93–1.78)	0.13	1.17(0.74–1.85)	0.51	1.92(1.02–3.60)	**0.04**
rs2403557	0.286	0.253	0.242	1.19(0.84–1.69)	0.33	1.23(0.79–1.93)	0.36	1.31(0.57–3.03)	0.53
rs6485345	0.292	0.211	0.250	1.26(0.87–1.81)	0.22	1.24(0.79–1.93)	0.35	1.81(0.68–4.78)	0.24
rs16910295	0.173	0.134	0.105	1.64(1.02–2.63)	0.04	1.52(0.91–2.52)	0.11	․	․
rs1038908	0.097	0.158	0.145	0.70(0.42–1.16)	0.17	0.70(0.42–1.16)	0.17	․	․
rs12295349	0.177	0.194	0.169	1.03(0.68–1.57)	0.88	1.15(0.72–1.83)	0.57	0.55(0.14–2.23)	0.40
rs12288230	0.177	0.189	0.161	1.07(0.71–1.63)	0.74	1.21(0.75–1.93)	0.44	0.55(0.14–2.23)	0.40
rs16910294	0.190	0.143	0.145	1.39(0.91–2.14)	0.13	1.43(0.88–2.34)	0.15	1.79(0.45–7.05)	0.41
rs7113678	0.102	0.107	0.105	0.90(0.54–1.50)	0.68	0.84(0.48–1.46)	0.53	2.31(0.20–26.72)	0.50
rs12224675	0.183	0.128	0.185	1.02(0.67–1.56)	0.92	1.03(0.63–1.68)	0.91	0.99(0.26–3.74)	0.99
rs7109243	0.438	0.474	0.508	0.82(0.60–1.13)	0.22	0.72(0.44–1.18)	0.20	0.83(0.49–1.40)	0.48
rs4757595	0.058	0.102	0.113	0.58(0.32–1.04)	0.07	0.58(0.32–1.04)	0.07	․	․
rs11022104	0.230	0.281	0.250	0.93(0.65–1.34)	0.69	1.07(0.69–1.68)	0.76	0.53(0.21–1.32)	0.17
rs12225759	0.442	0.459	0.492	0.85(0.60–1.19)	0.33	0.76(0.46–1.26)	0.28	0.88(0.50–1.56)	0.67
rs10765917	0.385	0.357	0.393	0.98(0.70–1.36)	0.90	0.90(0.57–1.42)	0.65	1.15(0.60–2.23)	0.68
rs6416121	0.058	0.102	0.097	0.66(0.37–1.20)	0.17	0.66(0.37–1.20)	0.17	․	․
rs7478946	0.469	0.454	0.435	1.17(0.83–1.63)	0.37	1.07(0.65–1.77)	0.79	1.45(0.81–2.60)	0.22
rs4757519	0.336	0.311	0.363	0.94(0.69–1.28)	0.69	0.83(0.53–1.30)	0.42	1.11(0.58–2.11)	0.75
rs7395522	0.177	0.235	0.153	1.05(0.71–1.55)	0.80	1.13(0.71–1.81)	0.60	0.81(0.28–2.39)	0.71
rs16910272	0.230	0.148	0.177	1.34(0.90–1.99)	0.15	1.43(0.89–2.30)	0.14	1.40(0.46–4.22)	0.55
rs3750936	0.257	0.296	0.290	0.87(0.60–1.25)	0.44	0.89(0.57–1.39)	0.61	0.65(0.26–1.65)	0.36
rs4586138	0.155	0.102	0.153	1.08(0.69–1.70)	0.73	1.02(0.61–1.69)	0.95	2.29(0.44–12.06)	0.33
rs11022099	0.212	0.209	0.208	1.04(0.70–1.55)	0.86	1.08(0.69–1.71)	0.74	0.81(0.24–2.78)	0.74
rs6485328	0.248	0.281	0.194	1.15(0.81–1.63)	0.43	1.32(0.84–2.08)	0.22	0.90(0.39–2.06)	0.80
rs7396140	0.447	0.490	0.435	1.03(0.77–1.38)	0.84	1.09(0.68–1.75)	0.71	0.99(0.59–1.66)	0.97
rs1552796	0.332	0.296	0.355	0.98(0.71–1.34)	0.88	0.85(0.54–1.32)	0.47	1.30(0.67–2.54)	0.44
rs7480815	0.385	0.388	0.410	0.93(0.69–1.26)	0.64	0.79(0.50–1.25)	0.32	1.12(0.63–1.99)	0.71
rs11022098	0.106	0.094	0.113	0.89(0.51–1.55)	0.67	0.89(0.51–1.55)	0.67	․	․
rs1994843	0.053	0.102	0.056	0.77(0.41–1.45)	0.42	0.77(0.41–1.45)	0.42	․	․
rs2087882	0.301	0.247	0.336	0.96(0.69–1.33)	0.80	0.87(0.56–1.36)	0.54	1.17(0.57–2.41)	0.67
rs2291599	0.243	0.270	0.169	1.25(0.88–1.79)	0.22	1.53(0.97–2.41)	0.07	0.86(0.36–2.05)	0.74
rs7396187	0.243	0.276	0.177	1.21(0.85–1.71)	0.29	1.53(0.97–2.41)	0.07	0.75(0.33–1.71)	0.49
rs1472190	0.301	0.255	0.339	0.96(0.69–1.33)	0.78	0.85(0.55–1.33)	0.48	1.22(0.59–2.51)	0.59
rs7480026	0.066	0.122	0.065	0.84(0.48–1.49)	0.55	0.85(0.47–1.52)	0.57	0.55(0.02–20.49)	0.75
rs7480000	0.407	0.398	0.344	1.17(0.85–1.61)	0.33	1.24(0.79–1.96)	0.36	1.22(0.66–2.25)	0.52
rs11022095	0.350	0.320	0.344	1.04(0.74–1.45)	0.84	1.06(0.68–1.65)	0.81	1.01(0.50–2.05)	0.98
rs1472189	0.150	0.115	0.169	0.96(0.60–1.51)	0.85	0.89(0.54–1.47)	0.66	1.96(0.28–13.53)	0.50
DKK3_B1_ht1	0.420	0.469	0.371	1.11(0.80–1.55)	0.54	1.23(0.76–1.98)	0.40	1.03(0.56–1.90)	0.92
DKK3_B1_ht2	0.102	0.122	0.121	0.87(0.53–1.44)	0.59	0.91(0.53–1.55)	0.72	0.43(0.05–3.68)	0.44
DKK3_B1_ht3	0.075	0.087	0.065	1.02(0.55–1.88)	0.95	1.02(0.55–1.88)	0.95	․	․
DKK3_B1_ht4	0.035	0.077	0.089	0.52(0.27–1.00)	**0.05**	0.54(0.27–1.06)	0.07	․	․
DKK3_B2_ht1	0.447	0.439	0.516	0.82(0.59–1.14)	0.24	0.73(0.44–1.21)	0.22	0.83(0.47–1.46)	0.51
DKK3_B2_ht2	0.243	0.219	0.105	1.77(1.17–2.67)	**0.007**	2.27(1.42–3.64)	**0.0007**	0.75(0.22–2.55)	0.64
DKK3_B2_ht3	0.137	0.168	0.218	0.70(0.46–1.06)	0.10	0.75(0.46–1.21)	0.24	0.27(0.07–1.02)	**0.05**
DKK3_B2_ht4	0.124	0.102	0.097	1.25(0.75–2.07)	0.40	1.36(0.78–2.35)	0.28	0.41(0.05–3.44)	0.41
DKK3_B3_ht1	0.274	0.265	0.234	1.13(0.81–1.59)	0.46	1.13(0.72–1.76)	0.60	1.35(0.62–2.94)	0.45
DKK3_B3_ht2	0.177	0.189	0.161	1.07(0.71–1.63)	0.74	1.21(0.75–1.93)	0.44	0.55(0.14–2.23)	0.40
DKK3_B3_ht3	0.173	0.122	0.185	0.96(0.63–1.47)	0.84	0.94(0.58–1.55)	0.82	0.99(0.26–3.71)	0.98
DKK3_B3_ht4	0.159	0.122	0.105	1.54(0.96–2.50)	0.08	1.41(0.84–2.37)	0.19	․	․
DKK3_B3_ht5	0.080	0.143	0.145	0.59(0.35–0.99)	0.05	0.59(0.35–0.99)	0.05	․	․
DKK3_B3_ht6	0.084	0.092	0.105	0.76(0.44–1.29)	0.31	0.67(0.37–1.21)	0.19	2.31(0.20–26.72)	0.50
DKK3_B4_ht1	0.381	0.357	0.379	1.00(0.72–1.40)	0.99	0.97(0.61–1.52)	0.88	1.09(0.56–2.12)	0.80
DKK3_B4_ht2	0.389	0.362	0.355	1.11(0.80–1.54)	0.54	1.13(0.72–1.79)	0.59	1.17(0.61–2.26)	0.64
DKK3_B4_ht3	0.164	0.179	0.153	1.06(0.69–1.61)	0.80	1.18(0.73–1.92)	0.49	0.55(0.14–2.23)	0.40
DKK3_B4_ht4	0.053	0.102	0.097	0.63(0.34–1.14)	0.13	0.63(0.34–1.14)	0.13	․	․
DKK3_B5_ht1	0.164	0.199	0.113	1.21(0.80–1.83)	0.36	1.36(0.83–2.21)	0.22	0.92(0.28–2.98)	0.88
DKK3_B5_ht2	0.168	0.158	0.153	1.13(0.74–1.73)	0.58	1.18(0.73–1.92)	0.50	0.90(0.22–3.61)	0.88
DKK3_B5_ht3	0.137	0.117	0.145	1.01(0.66–1.56)	0.96	0.96(0.57–1.62)	0.88	1.29(0.37–4.52)	0.69
DKK3_B5_ht4	0.080	0.061	0.097	0.83(0.45–1.53)	0.54	0.83(0.45–1.53)	0.54	․	․
DKK3_B5_ht5	0.137	0.092	0.129	1.10(0.69–1.76)	0.69	1.03(0.60–1.77)	0.91	2.29(0.44–12.06)	0.33
DKK3_B5_ht6	0.053	0.051	0.040	1.19(0.58–2.44)	0.63	1.24(0.58–2.65)	0.58	0.84(0.02–31.30)	0.92
DKK3_B5_ht7	0.049	0.087	0.048	0.83(0.42–1.61)	0.57	0.83(0.42–1.61)	0.57

Minor allele frequencies and P-values for logistic analyses of three alternative models (co-dominant, dominant and recessive models) controlling for age as covariate are shown.

Significant associations are shown in bold face (P-value<0.05).

### The association between DKK3 polymorphisms and clinical stage in prostate cancer group

In an analysis according to the clinical stage criteria, five SNPs (rs2403557, rs12295349, rs12288230, rs4586138, and rs7480000) were significant correlated with clinical stage in each model (OR 2.16, p=0.03 in the co-dominant model and OR 3.53, p=0.02 in the dominant; OR 2.70, p=0.05 in the dominant model; OR 2.78, p=0.04 in the dominant model; OR 2.25, p=0.04 in the co-dominant model and OR 8.71, p=0.01 in the recessive model; OR 2.72, p=0.006 in the co-dominant and OR 4.82, p=0.04 in the dominant model and OR 3.26, p=0.02 in the recessive model, respectively). In addition, two haplotypes (Block3_ht2 and Block5_ht5) were associated with clinical cancer stage (OR 2.78, p=0.04 in the dominant model; OR 2.53, p=0.02 in the co-dominant model and OR 8.71, p=0.01 in the recessive, respectively; Table-4, Supplementary Table-3).

**Table 4:** Logistic analysis of DKK3 polymorphisms according to clinical stage criteria.

SNPID	Minor Allele Frequency			Co-dominant		Dominant		Recessive	
	Metastatic (n=8)	Locally advanced (n=10)	Localized (n=252)	OR(95%CI)	P-value	OR(95%CI)	P-value	OR(95%CI)	P-value
rs2403557	0.500	0.350	0.254	2.16(1.07–4.33)	**0.03**	3.53(1.21–10.26)	**0.02**	1.69(0.36–7.95)	0.50
rs12295349	0.250	0.300	0.175	1.98(0.88–4.48)	0.10	2.70(1.02–7.15)	**0.05**	․	․
rs12288230	0.250	0.300	0.171	2.02(0.90–4.56)	0.09	2.78(1.05–7.36)	**0.04**	․	․
rs4586138	0.438	0.100	0.129	2.25(1.03–4.92)	**0.04**	2.00(0.74–5.38)	0.17	8.71(1.60–47.51)	**0.01**
rs7480000	0.750	0.500	0.378	2.72(1.33–5.56)	**0.006**	4.82(1.08–21.53)	**0.04**	3.26(1.17–9.11)	**0.02**
DKK3_B3_ht2	0.250	0.300	0.171	2.02(0.90–4.56)	0.09	2.78(1.05–7.36)	**0.04**	․	․
DKK3_B5_ht5	0.438	0.100	0.111	2.53(1.17–5.47)	**0.02**	2.43(0.90–6.56)	0.08	8.71(1.60–47.51)	**0.01**

Minor allele frequencies and P-values for logistic analyses of three alternative models (co-dominant, dominant and recessive models) controlling for age as covariate are shown. OR: odd ratio, Cl: confidence interval * Statistically significant at P < 0.05

**Supplementary Table 3:** Logistic analysis of DKK3 polymorphisms according to clinical stage criteria.

	Minor Allele Frequency			Co-dominant		Dominant		Recessive	
SNPID	Metastatic (n=8)	Locally advanced (n=10)	Localized (n=252)	OR(95%CI)	P-value	OR(95%CI)	P-value	OR(95%CI)	P-value
rs11022110	0.188	0.000	0.097	0.90(0.26–3.05)	0.86	0.92(0.26–3.28)	0.90	․	․
rs1493208	0.375	0.600	0.405	1.58(0.76–3.28)	0.22	2.61(0.73–9.33)	0.14	1.24(0.33–4.59)	0.75
rs751580	0.063	0.250	0.133	1.35(0.53–3.46)	0.53	1.55(0.55–4.35)	0.41	․	․
rs16910327	0.063	0.250	0.133	1.29(0.50–3.32)	0.60	1.11(0.38–3.26)	0.85	4.70(0.37–59.48)	0.23
rs3750940	0.125	0.100	0.145	0.73(0.25–2.14)	0.56	0.76(0.24–2.39)	0.64	․	․
rs11544817	0.063	0.250	0.129	1.38(0.55–3.45)	0.49	1.67(0.59–4.69)	0.34	․	․
rs2896594	0.125	0.100	0.139	0.75(0.25–2.22)	0.60	0.79(0.25–2.48)	0.68	․	․
rs7104941	0.063	0.100	0.126	0.60(0.17–2.06)	0.42	0.61(0.17–2.20)	0.45	․	․
rs3750938	0.313	0.400	0.311	1.30(0.62–2.71)	0.48	1.39(0.52–3.70)	0.51	1.41(0.30–6.67)	0.66
rs7107048	0.000	0.050	0.103	0.27(0.04–1.96)	0.19	0.25(0.03–1.99)	0.19	․	․
rs7478978	0.000	0.050	0.113	0.21(0.03–1.59)	0.13	0.20(0.03–1.58)	0.13	․	․
rs12421658	0.125	0.250	0.167	1.26(0.53–2.99)	0.60	1.56(0.57–4.21)	0.39	․	․
rs16910308	0.188	0.250	0.203	1.13(0.49–2.60)	0.78	1.08(0.40–2.88)	0.88	1.63(0.18–14.46)	0.66
rs11022105	0.313	0.500	0.394	1.14(0.56–2.36)	0.71	0.87(0.32–2.35)	0.79	1.90(0.58–6.29)	0.29
rs6485351	0.125	0.400	0.220	1.39(0.66–2.96)	0.39	1.70(0.64–4.49)	0.29	0.99(0.12–8.31)	1.00
rs7116879	0.188	0.250	0.219	1.03(0.44–2.41)	0.94	0.97(0.36–2.59)	0.95	1.54(0.18–13.52)	0.70
rs988666	0.563	0.400	0.397	1.38(0.70–2.74)	0.35	2.00(0.64–6.28)	0.23	1.10(0.31–3.91)	0.88
rs2403557	0.500	0.350	0.254	2.16(1.07–4.33)	**0.03**	3.53(1.21–10.26)	**0.02**	1.69(0.36–7.95)	0.50
rs6485345	0.438	0.150	0.253	1.18(0.54–2.59)	0.67	1.25(0.48–3.26)	0.65	1.13(0.14–9.04)	0.91
rs16910295	0.063	0.250	0.143	1.21(0.47–3.12)	0.69	1.35(0.48–3.80)	0.57	․	․
rs1038908	0.063	0.050	0.135	0.38(0.08–1.72)	0.21	0.38(0.08–1.72)	0.21	․	․
rs12295349	0.250	0.300	0.175	1.98(0.88–4.48)	0.10	2.70(1.02–7.15)	**0.05**	․	․
rs12288230	0.250	0.300	0.171	2.02(0.90–4.56)	0.09	2.78(1.05–7.36)	**0.04**	․	․
rs16910294	0.063	0.250	0.165	1.02(0.42–2.52)	0.96	1.20(0.43–3.37)	0.72	․	․
rs7113678	0.000	0.150	0.105	0.67(0.20–2.29)	0.53	0.71(0.19–2.60)	0.60	․	․
rs12224675	0.500	0.050	0.159	1.69(0.77–3.73)	0.19	1.92(0.73–5.08)	0.19	1.80(0.22–15.05)	0.59
rs7109243	0.813	0.400	0.460	1.70(0.85–3.37)	0.13	2.13(0.59–7.68)	0.25	1.94(0.70–5.37)	0.20
rs4757595	0.000	0.000	0.091	․	․	․	․	․	․
rs11022104	0.188	0.300	0.252	1.05(0.47–2.35)	0.91	1.32(0.51–3.46)	0.57	․	․
rs12225759	0.188	0.450	0.470	0.50(0.23–1.07)	0.07	0.39(0.15–1.02)	0.06	0.52(0.11–2.37)	0.39
rs10765917	0.188	0.450	0.382	0.74(0.36–1.55)	0.43	0.68(0.26–1.79)	0.43	0.70(0.15–3.30)	0.65
rs6416121	0.000	0.000	0.087	․	․	․	․	․	․
rs7478946	0.563	0.550	0.446	1.78(0.86–3.70)	0.12	3.64(0.80–16.54)	0.10	1.45(0.45–4.67)	0.53
rs4757519	0.313	0.350	0.329	0.99(0.50–1.95)	0.97	0.88(0.34–2.29)	0.78	1.24(0.34–4.54)	0.75
rs7395522	0.188	0.300	0.190	1.47(0.67–3.22)	0.34	2.05(0.78–5.40)	0.15	․	․
rs16910272	0.500	0.100	0.185	1.64(0.78–3.45)	0.19	1.59(0.60–4.21)	0.35	3.09(0.65–14.68)	0.16
rs3750936	0.000	0.250	0.288	0.37(0.14–1.01)	**0.05**	0.37(0.13–1.07)	0.07	․	․
rs4586138	0.438	0.100	0.129	2.25(1.03–4.92)	**0.04**	2.00(0.74–5.38)	0.17	8.71(1.60–47.51)	**0.01**
rs11022099	0.000	0.250	0.216	0.56(0.20–1.53)	0.26	0.58(0.20–1.68)	0.31	․	․
rs6485328	0.250	0.300	0.248	1.16(0.56–2.42)	0.69	1.37(0.52–3.58)	0.52	0.75(0.10–5.73)	0.78
rs7396140	0.250	0.550	0.466	0.86(0.45–1.64)	0.65	0.76(0.28–2.03)	0.58	0.89(0.28–2.82)	0.84
rs1552796	0.313	0.350	0.319	1.06(0.53–2.13)	0.87	0.92(0.35–2.39)	0.86	1.52(0.41–5.66)	0.53
rs7480815	0.313	0.350	0.390	0.79(0.39–1.58)	0.50	0.62(0.24–1.63)	0.33	0.99(0.27–3.59)	0.98
rs11022098	0.063	0.150	0.100	1.00(0.31–3.22)	1.00	1.00(0.31–3.22)	1.00	․	․
rs1994843	0.000	0.000	0.075	․	․	․	․	․	․
rs2087882	0.250	0.200	0.292	0.71(0.32–1.55)	0.39	0.69(0.26–1.83)	0.45	0.51(0.07–3.91)	0.51
rs2291599	0.313	0.300	0.234	1.47(0.71–3.02)	0.30	1.90(0.72–5.01)	0.19	0.91(0.12–6.95)	0.92
rs7396187	0.250	0.300	0.240	1.23(0.59–2.56)	0.58	1.48(0.57–3.87)	0.42	0.80(0.11–6.14)	0.83
rs1472190	0.250	0.200	0.296	0.71(0.32–1.55)	0.39	0.68(0.25–1.81)	0.44	0.52(0.07–4.03)	0.53
rs7480026	0.000	0.000	0.091	․	․	․	․	․	․
rs7480000	0.750	0.500	0.378	2.72(1.33–5.56)	**0.006**	4.82(1.08–21.53)	**0.04**	3.26(1.17–9.11)	**0.02**
rs11022095	0.125	0.450	0.340	0.81(0.38–1.71)	0.58	0.89(0.34–2.33)	0.81	0.45(0.06–3.56)	0.45
rs1472189	0.125	0.100	0.144	0.72(0.24–2.17)	0.56	0.51(0.14–1.84)	0.31	6.07(0.64–57.40)	0.12
DKK3_B1_ht1	0.438	0.650	0.417	1.90(0.91–3.95)	0.09	2.38(0.67–8.49)	0.18	2.23(0.73–6.75)	0.16
DKK3_B1_ht2	0.063	0.250	0.111	1.66(0.63–4.38)	0.30	1.86(0.66–5.26)	0.24	․	․
DKK3_B1_ht3	0.000	0.100	0.077	0.66(0.14–3.03)	0.59	0.66(0.14–3.03)	0.59	․	․
DKK3_B1_ht4	0.125	0.000	0.063	0.96(0.22–4.19)	0.95	0.97(0.22–4.37)	0.97	․	․
DKK3_B2_ht1	0.688	0.300	0.458	1.05(0.51–2.14)	0.90	0.95(0.32–2.81)	0.93	1.21(0.38–3.84)	0.75
DKK3_B2_ht2	0.188	0.250	0.202	1.13(0.49–2.61)	0.78	1.08(0.40–2.90)	0.88	1.63(0.18–14.49)	0.66
DKK3_B2_ht3	0.125	0.250	0.167	1.26(0.53–2.99)	0.60	1.56(0.57–4.21)	0.39	․	․
DKK3_B2_ht4	0.000	0.050	0.113	0.21(0.03–1.59)	0.13	0.20(0.03–1.58)	0.13	․	․
DKK3_B3_ht1	0.188	0.200	0.264	0.72(0.32–1.63)	0.43	0.51(0.18–1.47)	0.21	1.36(0.28–6.49)	0.70
DKK3_B3_ht2	0.250	0.300	0.171	2.02(0.90–4.56)	0.09	2.78(1.05–7.36)	**0.04**	․	․
DKK3_B3_ht3	0.438	0.050	0.155	1.47(0.65–3.31)	0.35	1.56(0.58–4.20)	0.38	1.81(0.22–15.12)	0.58
DKK3_B3_ht4	0.000	0.250	0.135	1.03(0.38–2.81)	0.96	1.12(0.38–3.32)	0.84	․	․
DKK3_B3_ht5	0.063	0.050	0.121	0.43(0.10–1.95)	0.27	0.43(0.10–1.95)	0.27	․	․
DKK3_B3_ht6	0.000	0.150	0.091	0.76(0.22–2.57)	0.66	0.81(0.22–3.03)	0.76	․	․
DKK3_B4_ht1	0.188	0.450	0.377	0.77(0.37–1.60)	0.48	0.71(0.27–1.86)	0.48	0.73(0.16–3.44)	0.69
DKK3_B4_ht2	0.625	0.250	0.367	1.28(0.63–2.60)	0.50	1.03(0.38–2.76)	0.96	2.17(0.66–7.07)	0.20
DKK3_B4_ht3	0.188	0.300	0.161	1.83(0.81–4.16)	0.15	2.44(0.93–6.43)	0.07	․	․
DKK3_B4_ht4	0.000	0.000	0.085	․	․	․	․	․	․
DKK3_B5_ht1	0.125	0.300	0.163	1.50(0.66–3.41)	0.34	1.98(0.74–5.25)	0.17	․	․
DKK3_B5_ht2	0.000	0.250	0.165	0.82(0.30–2.19)	0.69	0.89(0.30–2.62)	0.83	․	․
DKK3_B5_ht3	0.125	0.000	0.135	0.41(0.10–1.69)	0.22	0.41(0.09–1.80)	0.24	․	․
DKK3_B5_ht4	0.000	0.100	0.077	0.59(0.13–2.75)	0.50	0.59(0.13–2.75)	0.50	․	․
DKK3_B5_ht5	0.438	0.100	0.111	2.53(1.17–5.47)	**0.02**	2.43(0.90–6.56)	0.08	8.71(1.60–47.51)	**0.01**
DKK3_B5_ht6	0.063	0.000	0.052	0.54(0.07–3.98)	0.54	0.54(0.07–4.11)	0.55	․	․
DKK3_B5_ht7	0.000	0.000	0.065	․	․	․	․	․	․

Minor allele frequencies and P-values for logistic analyses of three alternative models (co-dominant, dominant and recessive models) controlling for age as covariate are shown.

Significant associations are shown in bold face (P-value<0.05).

### The association between DKK3 polymorphisms and pathologic stage in prostate cancer group

The pathological cancer stage was found to be associated with 9 SNPs (rs751580, rs11544817, rs4757519, rs1552796, rs11022098, rs2087882, rs1472190, rs11022095, and rs1472189) and one haplotype (Block1_ht2) (OR 2.08, p=0.01; OR 1.82, p=0.05; OR 1.64, p=0.009; OR 1.83, p=0.02; OR 1.88, p=0.05; OR 1.74, p=0.04; OR 1.46, p=0.05; OR 1.70, p=0.05; OR 2.66, p=0.0005; OR 1.93, p=0.04, respectively; Table-5, Supplementary Table-4). One SNP (rs1994843) and one haplotype (Block5_ht7) exhibited a protective effect from pathological cancer stage in both co-dominant and dominant model (OR 0.36, p=0.02; OR 0.33, p=0.01). Among 9 SNPS, 2 SNPs (rs4757519, rs1472189) exhibited a markedly significant association with greater aggressiveness.

**Table 5:** Logistic analysis of DKK3 polymorphisms according to pathological stage criteria.

SNPID	Minor Allele Frequency		Co-dominant		Dominant		Recessive	
	Locally advanced (n=100)	Localized (n=152)	OR(95%CI)	P-value	OR(95%CI)	P-value	OR(95%CI)	P-value
rs751580	0.180	0.107	1.90(1.11–3.23)	**0.02** *	2.08(1.16–3.72)	**0.01** *	1.63(0.23–11.79)	0.63
rs11544817	0.170	0.107	1.72(1.02–2.92)	**0.04** *	1.82(1.01–3.29)	**0.05** *	2.37(0.39–14.51)	0.35
rs4757519	0.418	0.290	1.64(1.13–2.38)	**0.009** *	1.85(1.09–3.14)	**0.02** *	2.13(1.02–4.44)	0.04*
rs1552796	0.397	0.290	1.54(1.06–2.23)	**0.02** *	1.83(1.08–3.09)	**0.02** *	1.67(0.79–3.54)	0.18
rs11022098	0.144	0.084	1.88(1.01–3.52)	**0.05** *	1.88(1.01–3.52)	**0.05** *	․	․
rs1994843	0.041	0.097	0.36(0.16–0.82)	**0.02** *	0.36(0.16–0.82)	**0.02** *	․	․
rs2087882	0.356	0.253	1.54(1.05–2.26)	**0.03** *	1.74(1.03–2.92)	**0.04** *	1.87(0.82–4.26)	0.13
rs1472190	0.351	0.263	1.46(1.00–2.14)	**0.05** *	1.55(0.92–2.59)	0.10	1.93(0.85–4.37)	0.12
rs11022095	0.387	0.304	1.44(0.98–2.12)	0.06	1.70(1.00–2.88)	**0.05** *	1.40(0.63–3.11)	0.40
rs1472189	0.216	0.101	2.66(1.54–4.59)	**0.0005** *	2.61(1.47–4.62)	**0.001** *	․	․
DKK3_B1_ht2	0.155	0.087	2.00(1.12–3.58)	**0.02** *	1.93(1.04–3.55)	**0.04** *	․	․
DKK3_B5_ht7	0.036	0.090	0.33(0.14–0.80)	**0.01** *	0.33(0.14–0.80)	**0.01** *	․	․

Minor allele frequencies and P-values for logistic analyses of three alternative models (co-dominant, dominant and recessive models) controlling for age as covariate are shown. OR: odd ratio, CI: confidence interval

* Statistically significant at P < 0.05

**Supplementary Table 4:** Logistic analysis of DKK3 polymorphisms according to pathological stage criteria.

	Minor Allele Frequency		Co-dominant		Dominant		Recessive	
SNPID	Locally advanced (n=97)	Localized (n=150)	OR(95%CI)	P-value	OR(95%CI)	P-value	OR(95%CI)	P-value
rs11022110	0.082	0.107	0.76(0.40–1.45)	0.41	0.80(0.41–1.57)	0.52	․	․
rs1493208	0.428	0.393	1.20(0.81–1.78)	0.37	1.34(0.77–2.34)	0.30	1.12(0.53–2.35)	0.77
rs751580	0.180	0.107	1.90(1.11–3.23)	**0.02**	2.08(1.16–3.72)	**0.01**	1.63(0.23–11.79)	0.63
rs16910327	0.103	0.143	0.68(0.38–1.21)	0.19	0.59(0.32–1.11)	0.10	2.67(0.24–30.24)	0.43
rs3750940	0.124	0.150	0.79(0.46–1.36)	0.39	0.80(0.44–1.44)	0.45	0.47(0.05–4.64)	0.52
rs11544817	0.170	0.107	1.72(1.02–2.92)	**0.04**	1.82(1.01–3.29)	**0.05**	2.37(0.39–14.51)	0.35
rs2896594	0.124	0.137	0.89(0.51–1.53)	0.66	0.91(0.50–1.66)	0.77	0.48(0.05–4.80)	0.54
rs7104941	0.113	0.122	0.92(0.53–1.62)	0.78	1.05(0.57–1.95)	0.88	․	․
rs3750938	0.325	0.292	1.19(0.80–1.77)	0.39	1.20(0.72–2.00)	0.50	1.40(0.58–3.42)	0.46
rs7107048	0.098	0.103	0.97(0.54–1.71)	0.90	1.07(0.55–2.08)	0.84	0.36(0.04–3.30)	0.37
rs7478978	0.103	0.110	0.95(0.52–1.73)	0.86	1.03(0.54–1.94)	0.93	․	․
rs12421658	0.165	0.173	0.96(0.59–1.55)	0.86	0.88(0.50–1.54)	0.65	1.60(0.39–6.59)	0.51
rs16910308	0.175	0.211	0.77(0.48–1.24)	0.28	0.78(0.46–1.35)	0.38	0.47(0.09–2.37)	0.36
rs11022105	0.366	0.409	0.81(0.55–1.21)	0.31	0.74(0.43–1.27)	0.28	0.84(0.38–1.84)	0.65
rs6485351	0.237	0.227	1.08(0.71–1.64)	0.73	1.11(0.66–1.88)	0.69	1.02(0.35–2.98)	0.97
rs7116879	0.186	0.232	0.74(0.46–1.17)	0.20	0.75(0.44–1.28)	0.29	0.42(0.08–2.06)	0.28
rs988666	0.433	0.373	1.27(0.88–1.84)	0.20	1.22(0.72–2.09)	0.46	1.67(0.84–3.32)	0.14
rs2403557	0.250	0.252	1.00(0.66–1.53)	1.00	0.98(0.58–1.65)	0.94	1.08(0.37–3.16)	0.89
rs6485345	0.268	0.245	1.11(0.72–1.69)	0.64	0.97(0.58–1.63)	0.90	2.22(0.74–6.63)	0.15
rs16910295	0.129	0.154	0.83(0.48–1.44)	0.51	0.82(0.46–1.47)	0.50	0.81(0.07–9.11)	0.86
rs1038908	0.134	0.130	1.14(0.63–2.06)	0.67	1.14(0.63–2.06)	0.67	․	․
rs12295349	0.170	0.170	1.03(0.63–1.69)	0.90	1.14(0.66–1.98)	0.64	0.34(0.04–2.98)	0.33
rs12288230	0.170	0.167	1.06(0.64–1.73)	0.83	1.18(0.68–2.04)	0.57	0.34(0.04–2.98)	0.33
rs16910294	0.134	0.187	0.70(0.42–1.16)	0.16	0.66(0.37–1.18)	0.16	0.60(0.11–3.17)	0.55
rs7113678	0.134	0.093	1.46(0.81–2.63)	0.21	1.51(0.81–2.81)	0.19	1.24(0.08–20.34)	0.88
rs12224675	0.139	0.164	0.77(0.46–1.30)	0.33	0.82(0.46–1.46)	0.50	0.27(0.03–2.40)	0.24
rs7109243	0.448	0.450	1.00(0.69–1.45)	0.99	1.10(0.63–1.93)	0.74	0.89(0.47–1.71)	0.73
rs4757595	0.067	0.103	0.62(0.31–1.27)	0.19	0.62(0.31–1.27)	0.19	․	․
rs11022104	0.216	0.260	0.83(0.54–1.28)	0.39	0.90(0.53–1.53)	0.70	0.40(0.11–1.49)	0.17
rs12225759	0.469	0.467	1.02(0.68–1.52)	0.93	0.96(0.53–1.74)	0.89	1.11(0.57–2.16)	0.76
rs10765917	0.407	0.362	1.21(0.82–1.78)	0.34	1.22(0.71–2.08)	0.47	1.39(0.66–2.94)	0.39
rs6416121	0.062	0.103	0.57(0.28–1.18)	0.13	0.57(0.28–1.18)	0.13	․	․
rs7478946	0.469	0.440	1.18(0.80–1.74)	0.41	1.68(0.92–3.07)	0.09	0.82(0.41–1.63)	0.57
rs4757519	0.418	0.290	1.64(1.13–2.38)	**0.009**	1.85(1.09–3.14)	**0.02**	2.13(1.02–4.44)	0.04
rs7395522	0.149	0.207	0.71(0.44–1.15)	0.16	0.79(0.45–1.38)	0.40	0.16(0.02–1.26)	0.08
rs16910272	0.165	0.200	0.77(0.49–1.23)	0.28	0.77(0.44–1.35)	0.36	0.53(0.14–2.06)	0.36
rs3750936	0.263	0.293	0.87(0.57–1.34)	0.54	0.93(0.55–1.55)	0.77	0.57(0.18–1.85)	0.35
rs4586138	0.113	0.147	0.72(0.42–1.25)	0.25	0.74(0.40–1.35)	0.32	0.34(0.04–3.13)	0.34
rs11022099	0.214	0.201	1.13(0.71–1.79)	0.62	1.17(0.69–2.00)	0.56	0.96(0.22–4.12)	0.96
rs6485328	0.206	0.263	0.75(0.49–1.14)	0.18	0.72(0.42–1.22)	0.22	0.61(0.21–1.78)	0.36
rs7396140	0.428	0.467	0.91(0.64–1.29)	0.58	0.79(0.46–1.36)	0.39	1.00(0.54–1.85)	1.00
rs1552796	0.397	0.290	1.54(1.06–2.23)	**0.02**	1.83(1.08–3.09)	**0.02**	1.67(0.79–3.54)	0.18
rs7480815	0.438	0.383	1.20(0.84–1.71)	0.32	1.21(0.71–2.08)	0.48	1.38(0.72–2.65)	0.33
rs11022098	0.144	0.084	1.88(1.01–3.52)	**0.05**	1.88(1.01–3.52)	**0.05**	․	․
rs1994843	0.041	0.097	0.36(0.16–0.82)	**0.02**	0.36(0.16–0.82)	**0.02**	․	․
rs2087882	0.356	0.253	1.54(1.05–2.26)	**0.03**	1.74(1.03–2.92)	**0.04**	1.87(0.82–4.26)	0.13
rs2291599	0.206	0.247	0.82(0.54–1.25)	0.36	0.81(0.48–1.37)	0.42	0.68(0.23–2.01)	0.48
rs7396187	0.211	0.247	0.85(0.56–1.29)	0.43	0.81(0.48–1.37)	0.43	0.82(0.29–2.27)	0.70
rs1472190	0.351	0.263	1.46(1.00–2.14)	0.05	1.55(0.92–2.59)	0.10	1.93(0.85–4.37)	0.12
rs7480026	0.062	0.107	0.53(0.26–1.08)	0.08	0.54(0.26–1.11)	0.09	․	․
rs7480000	0.340	0.413	0.73(0.51–1.07)	0.10	0.67(0.40–1.14)	0.14	0.65(0.31–1.35)	0.25
rs11022095	0.387	0.304	1.44(0.98–2.12)	0.06	1.70(1.00–2.88)	**0.05**	1.40(0.63–3.11)	0.40
rs1472189	0.216	0.101	2.66(1.54–4.59)	**0.0005**	2.61(1.47–4.62)	**0.001**	․	․
DKK3_B1_ht1	0.438	0.403	1.20(0.81–1.76)	0.36	1.30(0.74–2.27)	0.36	1.19(0.59–2.42)	0.62
DKK3_B1_ht2	0.155	0.087	2.00(1.12–3.58)	**0.02**	1.93(1.04–3.55)	**0.04**	․	․
DKK3_B1_ht3	0.067	0.073	0.90(0.43–1.89)	0.78	0.90(0.43–1.89)	0.78	․	․
DKK3_B1_ht4	0.041	0.073	0.57(0.25–1.32)	0.19	0.58(0.24–1.37)	0.21	․	․
DKK3_B2_ht1	0.474	0.450	1.10(0.75–1.62)	0.62	1.17(0.65–2.10)	0.60	1.09(0.56–2.09)	0.80
DKK3_B2_ht2	0.175	0.210	0.78(0.48–1.25)	0.30	0.79(0.46–1.36)	0.40	0.47(0.09–2.38)	0.36
DKK3_B2_ht3	0.165	0.173	0.96(0.59–1.55)	0.86	0.88(0.50–1.54)	0.65	1.60(0.39–6.59)	0.51
DKK3_B2_ht4	0.103	0.110	0.95(0.52–1.73)	0.86	1.03(0.54–1.94)	0.93	․	․
DKK3_B3_ht1	0.289	0.263	1.12(0.76–1.65)	0.57	1.22(0.73–2.04)	0.45	0.99(0.41–2.39)	0.98
DKK3_B3_ht2	0.170	0.167	1.06(0.64–1.73)	0.83	1.18(0.68–2.04)	0.57	0.34(0.04–2.98)	0.33
DKK3_B3_ht3	0.134	0.157	0.78(0.46–1.32)	0.35	0.83(0.46–1.49)	0.53	0.28(0.03–2.42)	0.24
DKK3_B3_ht4	0.119	0.143	0.83(0.47–1.45)	0.51	0.82(0.45–1.49)	0.50	0.82(0.07–9.16)	0.87
DKK3_B3_ht5	0.124	0.113	1.21(0.66–2.22)	0.55	1.21(0.66–2.22)	0.55	․	․
DKK3_B3_ht6	0.119	0.080	1.46(0.78–2.71)	0.24	1.52(0.78–2.93)	0.22	1.24(0.08–20.34)	0.88
DKK3_B4_ht1	0.397	0.363	1.15(0.78–1.69)	0.47	1.11(0.65–1.88)	0.71	1.40(0.66–2.97)	0.38
DKK3_B4_ht2	0.376	0.377	0.97(0.66–1.42)	0.87	0.88(0.52–1.49)	0.63	1.14(0.54–2.41)	0.72
DKK3_B4_ht3	0.155	0.157	1.03(0.62–1.70)	0.91	1.14(0.65–2.02)	0.64	0.34(0.04–2.98)	0.33
DKK3_B4_ht4	0.057	0.103	0.52(0.25–1.10)	0.09	0.52(0.25–1.10)	0.09	․	․
DKK3_B5_ht1	0.134	0.180	0.75(0.45–1.23)	0.25	0.81(0.45–1.43)	0.46	0.21(0.03–1.74)	0.15
DKK3_B5_ht2	0.144	0.173	0.83(0.50–1.38)	0.48	0.89(0.50–1.56)	0.67	0.31(0.04–2.67)	0.28
DKK3_B5_ht3	0.124	0.133	0.92(0.55–1.54)	0.74	0.95(0.52–1.75)	0.87	0.61(0.12–3.22)	0.56
DKK3_B5_ht4	0.108	0.063	1.81(0.91–3.60)	0.09	1.81(0.91–3.60)	0.09	․	․
DKK3_B5_ht5	0.093	0.137	0.64(0.36–1.13)	0.12	0.63(0.33–1.19)	0.16	0.34(0.04–3.13)	0.34
DKK3_B5_ht6	0.052	0.053	0.96(0.43–2.15)	0.92	0.84(0.36–2.00)	0.70	․	․
DKK3_B5_ht7	0.036	0.090	0.33(0.14–0.80)	**0.01**	0.33(0.14–0.80)	**0.01**	․	․

Minor allele frequencies and P-values for logistic analyses of three alternative models (co-dominant, dominant and recessive models) controlling for age as covariate are shown.

Significant associations are shown in bold face (P-value<0.05)

### The association between DKK3 polymorphisms and Gleason score in prostate cancer group

When we stratified the patients according to Gleason score, we observed that the association of prostate cancer risk with one SNP (rs7478946) and one haplotype (Block4_ht2) was strongest among patients with high compared to low grade Gleason scores (OR 2.32, p=0.01 in the dominant model; OR 2.23, p=0.05 in the recessive model, respectively; Table-6, Supplementary Table-5).

**Table 6:** Logistic analysis of DKK3 polymorphisms according to Gleason score criteria.

	Minor Allele Frequency			Co-dominant		Dominant		Recessive	
SNPID	High grade (n=39)	Intermediate (n=202)	Low grade (n=29)	OR(95%CI)	P-value	OR(95%CI)	P-value	OR(95%CI)	P-value
rs7478946	0.458	0.379	0.454	1.37(0.90–2.07)	0.14	2.32(1.22–4.40)	0.01	0.85(0.41–1.74)	0.65
DKK3_B4_ht2	0.410	0.364	0.345	1.17(0.78–1.75)	0.46	0.90(0.52–1.58)	0.72	2.23(1.01–4.91)	0.05

Minor allele frequencies and P-values for logistic analyses of three alternative models (co-dominant, dominant and recessive models) controlling for age as covariate are shown. **OR:** odd ratio, **CI:** confidence interval * Statistically significant at P < 0.05

**Supplementary Table 5:** Logistic analysis of DKK3 polymorphisms according to Gleason score criteria.

	Minor Allele Frequency			Co-dominant		Dominant		Recessive	
SNPID	High grade (n=39)	Intermediate (n=202)	Low grade (n=29)	OR(95%CI)	P-value	OR(95%CI)	P-value	OR(95%CI)	P-value
rs11022110	0.111	0.052	0.098	0.99(0.52–1.91)	0.98	1.10(0.55–2.20)	0.78	0.13(0.01–2.14)	0.15
rs1493208	0.401	0.500	0.411	0.80(0.53–1.22)	0.30	0.98(0.55–1.76)	0.94	0.47(0.21–1.03)	0.06
rs751580	0.136	0.121	0.135	1.13(0.64–1.98)	0.67	1.07(0.57–1.99)	0.84	2.32(0.34–15.84)	0.39
rs16910327	0.121	0.224	0.135	0.70(0.39–1.25)	0.22	0.68(0.36–1.27)	0.22	0.72(0.05–9.91)	0.81
rs3750940	0.144	0.207	0.144	0.60(0.34–1.07)	0.08	0.61(0.33–1.13)	0.12	0.27(0.03–2.24)	0.23
rs11544817	0.131	0.121	0.131	1.14(0.65–1.99)	0.64	0.99(0.52–1.88)	0.98	3.85(0.73–20.42)	0.11
rs2896594	0.139	0.190	0.139	0.65(0.37–1.15)	0.14	0.66(0.35–1.23)	0.19	0.29(0.04–2.34)	0.24
rs7104941	0.130	0.121	0.125	0.89(0.50–1.60)	0.70	0.87(0.46–1.66)	0.67	0.95(0.10–9.10)	0.96
rs3750938	0.333	0.276	0.316	0.93(0.61–1.41)	0.72	0.97(0.56–1.68)	0.92	0.75(0.29–1.93)	0.54
rs7107048	0.099	0.103	0.102	1.06(0.59–1.92)	0.84	1.02(0.50–2.05)	0.97	1.53(0.26–9.08)	0.64
rs7478978	0.094	0.207	0.111	0.68(0.36–1.26)	0.22	0.58(0.30–1.14)	0.12	2.68(0.26–27.99)	0.41
rs12421658	0.168	0.155	0.169	1.14(0.68–1.90)	0.62	1.04(0.57–1.87)	0.91	2.54(0.57–11.28)	0.22
rs16910308	0.209	0.172	0.204	1.12(0.69–1.82)	0.66	1.12(0.64–1.96)	0.70	1.30(0.29–5.79)	0.73
rs11022105	0.405	0.328	0.396	1.21(0.80–1.82)	0.37	1.15(0.65–2.04)	0.64	1.53(0.70–3.35)	0.28
rs6485351	0.228	0.155	0.224	1.40(0.89–2.21)	0.15	1.31(0.75–2.30)	0.35	2.74(0.91–8.27)	0.07
rs7116879	0.226	0.172	0.219	1.16(0.72–1.87)	0.55	1.17(0.67–2.04)	0.58	1.30(0.32–5.39)	0.72
rs988666	0.371	0.534	0.402	0.91(0.61–1.35)	0.63	0.85(0.48–1.50)	0.58	0.93(0.44–1.96)	0.85
rs2403557	0.241	0.310	0.265	1.23(0.80–1.89)	0.35	1.08(0.63–1.88)	0.78	2.44(0.92–6.44)	0.07
rs6485345	0.259	0.190	0.255	1.30(0.83–2.04)	0.25	1.24(0.72–2.15)	0.44	2.12(0.70–6.45)	0.19
rs16910295	0.142	0.155	0.145	1.05(0.60–1.84)	0.87	1.08(0.58–1.98)	0.82	0.82(0.09–7.83)	0.86
rs1038908	0.139	0.155	0.131	0.64(0.34–1.21)	0.17	0.64(0.34–1.21)	0.17	․	․
rs12295349	0.176	0.190	0.181	1.11(0.67–1.86)	0.68	1.25(0.70–2.21)	0.46	0.51(0.10–2.74)	0.43
rs12288230	0.173	0.172	0.178	1.19(0.71–1.98)	0.51	1.35(0.76–2.41)	0.31	0.51(0.10–2.74)	0.43
rs16910294	0.166	0.155	0.165	1.07(0.64–1.79)	0.79	1.14(0.63–2.07)	0.66	0.80(0.16–4.02)	0.79
rs7113678	0.104	0.052	0.104	1.61(0.87–3.00)	0.13	1.64(0.83–3.25)	0.15	2.58(0.25–26.98)	0.43
rs12224675	0.147	0.224	0.164	0.93(0.55–1.57)	0.79	1.03(0.57–1.88)	0.92	0.39(0.08–1.87)	0.24
rs7109243	0.455	0.552	0.469	0.86(0.58–1.26)	0.43	0.77(0.42–1.40)	0.39	0.88(0.46–1.69)	0.70
rs4757595	0.082	0.121	0.087	0.86(0.42–1.77)	0.68	0.86(0.42–1.77)	0.68	․	․
rs11022104	0.250	0.293	0.254	0.91(0.58–1.43)	0.69	0.95(0.55–1.65)	0.86	0.70(0.23–2.14)	0.53
rs12225759	0.465	0.483	0.463	0.88(0.58–1.33)	0.53	0.87(0.47–1.63)	0.67	0.81(0.40–1.64)	0.55
rs10765917	0.389	0.357	0.379	0.93(0.62–1.39)	0.71	0.85(0.48–1.50)	0.58	1.02(0.46–2.28)	0.96
rs6416121	0.077	0.121	0.083	0.87(0.42–1.81)	0.71	0.87(0.42–1.81)	0.71	․	․
rs7478946	0.458	0.379	0.454	1.37(0.90–2.07)	0.14	2.32(1.22–4.40)	0.01	0.85(0.41–1.74)	0.65
rs4757519	0.351	0.241	0.331	1.06(0.72–1.56)	0.77	1.24(0.71–2.14)	0.45	0.82(0.37–1.81)	0.62
rs7395522	0.196	0.155	0.193	1.22(0.76–1.96)	0.42	1.35(0.76–2.41)	0.30	0.94(0.25–3.54)	0.92
rs16910272	0.181	0.190	0.189	1.14(0.71–1.84)	0.59	1.09(0.61–1.94)	0.77	1.73(0.49–6.17)	0.40
rs3750936	0.270	0.362	0.280	0.79(0.50–1.25)	0.31	0.85(0.49–1.46)	0.55	0.46(0.15–1.43)	0.18
rs4586138	0.116	0.190	0.137	1.16(0.67–2.00)	0.60	1.13(0.61–2.12)	0.70	1.75(0.30–10.15)	0.53
rs11022099	0.206	0.268	0.213	0.83(0.51–1.36)	0.46	0.93(0.53–1.63)	0.80	0.31(0.07–1.30)	0.11
rs6485328	0.257	0.190	0.246	1.10(0.71–1.69)	0.68	1.29(0.74–2.25)	0.36	0.69(0.24–1.97)	0.49
rs7396140	0.460	0.500	0.461	0.91(0.63–1.31)	0.61	1.15(0.64–2.06)	0.65	0.63(0.33–1.21)	0.17
rs1552796	0.342	0.241	0.322	1.04(0.71–1.55)	0.83	1.16(0.67–2.01)	0.59	0.86(0.38–1.97)	0.72
rs7480815	0.408	0.310	0.388	1.03(0.70–1.50)	0.89	1.18(0.68–2.07)	0.56	0.84(0.41–1.72)	0.63
rs11022098	0.105	0.069	0.103	1.26(0.64–2.48)	0.51	1.26(0.64–2.48)	0.51	․	․
rs1994843	0.072	0.069	0.070	0.92(0.42–2.02)	0.84	0.92(0.42–2.02)	0.84	․	․
rs2087882	0.316	0.196	0.289	0.97(0.65–1.46)	0.89	1.17(0.68–2.03)	0.57	0.59(0.24–1.44)	0.24
rs2291599	0.240	0.190	0.235	1.18(0.76–1.82)	0.47	1.42(0.81–2.48)	0.22	0.70(0.23–2.11)	0.53
rs7396187	0.248	0.190	0.239	1.11(0.72–1.72)	0.62	1.32(0.76–2.30)	0.33	0.72(0.25–2.04)	0.53
rs1472190	0.317	0.224	0.293	0.93(0.62–1.40)	0.73	1.06(0.62–1.83)	0.83	0.61(0.25–1.50)	0.28
rs7480026	0.087	0.086	0.085	0.92(0.45–1.86)	0.81	0.92(0.44–1.91)	0.82	0.74(0.01–67.85)	0.90
rs7480000	0.376	0.393	0.390	1.20(0.81–1.79)	0.36	1.14(0.64–2.01)	0.66	1.57(0.75–3.31)	0.23
rs11022095	0.331	0.357	0.340	1.05(0.70–1.59)	0.80	0.84(0.48–1.46)	0.54	1.92(0.83–4.46)	0.13
rs1472189	0.155	0.052	0.144	1.50(0.86–2.63)	0.15	1.66(0.90–3.06)	0.11	0.87(0.09–8.24)	0.90
DKK3_B1_ht1	0.423	0.411	0.534	0.81(0.53–1.21)	0.30	0.82(0.46–1.48)	0.52	0.66(0.31–1.41)	0.28
DKK3_B1_ht2	0.128	0.116	0.086	1.28(0.70–2.34)	0.43	1.20(0.62–2.31)	0.59	4.24(0.40–44.36)	0.23
DKK3_B1_ht3	0.051	0.079	0.086	0.73(0.34–1.56)	0.41	0.73(0.34–1.56)	0.41	․	․
DKK3_B1_ht4	0.013	0.072	0.069	0.56(0.25–1.24)	0.15	0.62(0.27–1.43)	0.26	․	․
DKK3_B2_ht1	0.410	0.463	0.466	0.83(0.55–1.25)	0.38	0.67(0.37–1.24)	0.20	0.98(0.48–1.97)	0.95
DKK3_B2_ht2	0.205	0.208	0.172	1.12(0.69–1.82)	0.66	1.12(0.64–1.96)	0.71	1.31(0.29–5.81)	0.73
DKK3_B2_ht3	0.179	0.168	0.155	1.14(0.68–1.90)	0.62	1.04(0.57–1.87)	0.91	2.54(0.57–11.28)	0.22
DKK3_B2_ht4	0.128	0.094	0.207	0.68(0.36–1.26)	0.22	0.58(0.30–1.14)	0.12	2.68(0.26–27.99)	0.41
DKK3_B3_ht1	0.244	0.265	0.241	1.00(0.66–1.52)	1.00	0.80(0.46–1.39)	0.43	1.90(0.75–4.80)	0.18
DKK3_B3_ht2	0.205	0.173	0.172	1.19(0.71–1.98)	0.51	1.35(0.76–2.41)	0.31	0.51(0.10–2.74)	0.43
DKK3_B3_ht3	0.179	0.146	0.207	0.87(0.52–1.48)	0.61	0.95(0.52–1.74)	0.86	0.39(0.08–1.86)	0.24
DKK3_B3_ht4	0.115	0.136	0.155	0.85(0.48–1.51)	0.58	0.83(0.45–1.57)	0.57	0.82(0.09–7.86)	0.86
DKK3_B3_ht5	0.064	0.124	0.155	0.57(0.30–1.10)	0.09	0.57(0.30–1.10)	0.09	․	․
DKK3_B3_ht6	0.115	0.092	0.052	1.40(0.73–2.69)	0.31	1.39(0.67–2.86)	0.37	2.58(0.25–26.98)	0.43
DKK3_B4_ht1	0.346	0.381	0.362	0.93(0.62–1.39)	0.71	0.84(0.48–1.47)	0.55	1.05(0.47–2.36)	0.90
DKK3_B4_ht2	0.410	0.364	0.345	1.17(0.78–1.75)	0.46	0.90(0.52–1.58)	0.72	2.23(1.01–4.91)	0.05
DKK3_B4_ht3	0.154	0.168	0.172	0.96(0.57–1.61)	0.86	1.03(0.57–1.85)	0.93	0.51(0.10–2.74)	0.43
DKK3_B4_ht4	0.090	0.074	0.121	0.87(0.42–1.84)	0.72	0.87(0.42–1.84)	0.72	․	․
DKK3_B5_ht1	0.179	0.168	0.121	1.28(0.78–2.11)	0.33	1.42(0.78–2.58)	0.25	1.00(0.23–4.32)	1.00
DKK3_B5_ht2	0.167	0.153	0.224	0.84(0.50–1.43)	0.53	0.89(0.49–1.62)	0.70	0.45(0.08–2.39)	0.35
DKK3_B5_ht3	0.051	0.144	0.138	0.65(0.38–1.12)	0.12	0.54(0.28–1.05)	0.07	0.90(0.18–4.53)	0.90
DKK3_B5_ht4	0.090	0.079	0.052	1.28(0.60–2.71)	0.52	1.28(0.60–2.71)	0.52	․	․
DKK3_B5_ht5	0.179	0.106	0.138	1.29(0.73–2.26)	0.39	1.30(0.67–2.50)	0.44	1.75(0.30–10.15)	0.53
DKK3_B5_ht6	0.026	0.054	0.034	0.84(0.34–2.03)	0.69	0.81(0.32–2.09)	0.67	1.04(0.01–96.11)	0.99
DKK3_B5_ht7	0.064	0.062	0.052	1.11(0.49–2.54)	0.81	1.11(0.49–2.54)	0.81	․	․

Minor allele frequencies and P-values for logistic analyses of three alternative models (co-dominant, dominant and recessive models) controlling for age as covariate are shown.

Significant associations are shown in bold face (P-value<0.05)

## Discussion

SNPs are the most common polymorphisms in the genomes of many species. The definition of a SNP is a variation of the DNA sequence at a frequency larger than 1% of the allele of a population (5). In this study, we examined whether genetic variations in the DKK3 gene alter the risk of developing prostate cancer. A total of 53 SNPs located in the DKK3 gene were genotyped in 272 patients with prostate cancer and 173 control subjects with BPH. We found that three SNPs and two haplotypes were significantly associated with prostate cancer risk (p<0.05). Also, we found that two SNPs were markedly significantly associated with prostate cancer aggressiveness (P<0.001). These findings suggest that DKK3 gene polymorphisms may alter susceptibility to prostate cancer and could thus possibly be used as biomarkers for the disease and predictors for aggressivenessin patient with prostate cancer.

The Dickkopf (DKK) family consists of four genes (DKK1–4) and a DKK3-related gene (20). DKK3 is the most divergent member of the DKK family by DNA sequence, function, and evolution. Unlike the other DKK members, DKK3 does not modulate Wnt signaling (11) and shows no affinity to the Wnt co-receptor LRP5/6 and Kremen (21). Human DKK3 was proposed to function as a tumor suppressor, since its expression is down-regulated in many types of cancer cells (11). DKK3 down-regulation has been reported in endometrial cancer (22), lung cancer (23), gastrointestinal cancer (24), breast cancer (25), prostate cancer (26, 27), and renal carcinomas (28). In support of the hypothesis that DKK3 functions in prostate as a tumor suppressor, overexpression of DKK3 suppresses cell growth and the invasive capacity of prostate cancer cell lines (26, 27). Zenzmaier et al. reported that in normal prostate tissue the secreted glycoprotein DKK3 is expressed in the epithelial compartment but expression is lost in BPH and prostate cancer (29). DKK3 promotes fibroblast proliferation and myofibroblast differentiation and represents a potential therapeutic target for stromal remodeling in BPH and prostate cancer (30).

To our knowledge, one epidemiological investigation between DKK3 and prostate cancer have been reported. Zenzmaier et al. reported that DKK3 levels in seminal plasma were significantly elevated in biopsy-confirmed prostate cancer patients, because loss of expression seems to be counterbalanced by upregulation of DKK3 expression (31). In this study, we epidemiologically investigated whether SNPs of the DKK3 gene were related to the risk and aggressiveness of prostate cancer for the first time.

We note that this study has several limitations. Our sample size was relatively small for a case-control association study; it is limited in subgroup analysis. Therefore, the study requires further confirmation in much larger cohorts. However this study included a unique racial population, and the prostate cancer cohorts had a similar clinical characters of a previous Korean study (13) and the control group also had little selection bias due to their exclusion by biopsy. Although control group underwent prostate biopsy which reveals negative malignancy, all the men in the BPH group are potentially at risk for development of prostate cancer and may have latent prostate cancer at time of designation as controls, leading to disease misclassification. In addition we could not evaluate the effect of treatment related to BPH or prostate cancer such as medication or surgery in individuals. For many gene-exposure studies, a key limitation is the quality of the exposure information. Few studies have the ability to examine interactions between pesticide exposure and genetic risk factors for prostate cancer, thus replication of these findings may be difficult. Despite these limitations, the present study provides the first evidence of an important and novel association between DKK3 polymorphisms and the risk for prostate cancer, so will be a basis for future study.

## Conclusions

We have observed several positive interactions between DKK3 gene polymorphisms and the risk of prostate cancer in a Korean population for the first time. These findings could be helpful to diagnose and predict the prognosis of prostate cancer. We anticipate that patients' genomic data could be used in clinical practice to investigate associations between the risk of prostate cancer and polymorphisms in the DKK3 gene in the near future.

## References

[1] Jemal A, Siegel R, Ward E, Hao Y, Xu J, Thun MJ (2009). Cancer statistics, 2009. CA Cancer J Clin.

[2] Ferlay J, Autier P, Boniol M, Heanue M, Colombet M, Boyle P (2007). Estimates of the cancer incidence and mortality in Europe in 2006. Ann Oncol.

[3] Park SK, Sakoda LC, Kang D, Chokkalingam AP, Lee E, Shin HR (2006). Rising prostate cancer rates in South Korea. Prostate.

[4] Crawford ED (2009). Understanding the epidemiology, natural history, and key pathways involved in prostate cancer. Urology.

[5] Nakagawa H, Akamatsu S, Takata R, Takahashi A, Kubo M, Nakamura Y (2012). Prostate cancer genomics, biology, and risk assessment through genome-wide association studies. Cancer Sci.

[6] Zhang J, Dhakal IB, Greene G, Lang NP, Kadlubar FF (2010). Polymorphisms in hOGG1 and XRCC1 and risk of prostate cancer: effects modified by plasma antioxidants. Urology.

[7] Park K, Kim JH, Jeon HG, Byun SS, Lee E (2010). Influence of IGFBP3 gene polymorphisms on IGFBP3 serum levels and the risk of prostate cancer in low-risk Korean men. Urology.

[8] Klaus A, Birchmeier W (2008). Wnt signalling and its impact on development and cancer. Nat Rev Cancer.

[9] Polakis P (2012). Wnt signaling in cancer. Cold Spring Harb Perspect Biol.

[10] Kawano Y, Kypta R (2003). Secreted antagonists of the Wnt signalling pathway. J Cell Sci.

[11] Veeck J, Dahl E (2012). Targeting the Wnt pathway in cancer: the emerging role of Dickkopf-3. Biochim Biophys Acta.

[12] Lodygin D, Epanchintsev A, Menssen A, Diebold J, Hermeking H (2005). Functional epigenomics identifies genes frequently silenced in prostate cancer. Cancer Res.

[13] Song SY, Kim SR, Ahn G, Choi HY (2003). Pathologic characteristics of prostatic adenocarcinomas: a mapping analysis of Korean patients. Prostate Cancer Prostatic Dis.

[14] Oliphant A, Barker DL, Stuelpnagel JR, Chee MS (2002). BeadArray technology: enabling an accurate, cost-effective approach to high-throughput genotyping. Biotechniques.

[15] Morris JA, Gardner MJ (1988). Calculating confidence intervals for relative risks (odds ratios) and standardised ratios and rates. Br Med J (Clin Res Ed).

[16] Hedrick PW (1987). Gametic disequilibrium measures: proceed with caution. Genetics.

[17] Stephens M, Smith NJ, Donnelly P (2001). A new statistical method for haplotype reconstruction from population data. Am J Hum Genet.

[18] Nyholt DR (2004). A simple correction for multiple testing for single-nucleotide polymorphisms in linkage disequilibrium with each other. Am J Hum Genet.

[19] Menashe I, Rosenberg PS, Chen BE (2008). PGA: power calculator for case-control genetic association analyses. BMC Genet.

[20] Niehrs C (2006). Function and biological roles of the Dickkopf family of Wnt modulators. Oncogene.

[21] Mao J, Wang J, Liu B, Pan W, Farr GH, Flynn C (2001). Low-density lipoprotein receptor-related protein-5 binds to Axin and regulates the canonical Wnt signaling pathway. Mol Cell.

[22] Dellinger TH, Planutis K, Jandial DD, Eskander RN, Martinez ME, Zi X (2012). Expression of the Wnt antagonist Dickkopf-3 is associated with prognostic clinicopathologic characteristics and impairs proliferation and invasion in endometrial cancer. Gynecol Oncol.

[23] Nozaki I, Tsuji T, Iijima O, Ohmura Y, Andou A, Miyazaki M (2001). Reduced expression of REIC/Dkk-3 gene in non-small cell lung cancer. Int J Oncol.

[24] Yu J, Tao Q, Cheng YY, Lee KY, Ng SS, Cheung KF (2009). Promoter methylation of the Wnt/beta-catenin signaling antagonist Dkk-3 is associated with poor survival in gastric cancer. Cancer.

[25] Veeck J, Bektas N, Hartmann A, Kristiansen G, Heindrichs U, Kn?hel R (2008). Wnt signalling in human breast cancer: expression of the putative Wnt inhibitor Dickkopf-3 (DKK3) is frequently suppressed by promoter hypermethylation in mammary tumours. Breast Cancer Res.

[26] Abarzua F, Sakaguchi M, Takaishi M, Nasu Y, Kurose K, Ebara S (2005). Adenovirus-mediated overexpression of REIC/Dkk-3 selectively induces apoptosis in human prostate cancer cells through activation of c-Jun-NH2-kinase. Cancer Res.

[27] Kawano Y, Kitaoka M, Hamada Y, Walker MM, Waxman J, Kypta RM (2006). Regulation of prostate cell growth and morphogenesis by Dickkopf-3. Oncogene.

[28] Kurose K, Sakaguchi M, Nasu Y, Ebara S, Kaku H, Kariyama R (2004). Decreased expression of REIC/Dkk-3 in human renal clear cell carcinoma. J Urol.

[29] Zenzmaier C, Untergasser G, Hermann M, Dirnhofer S, Sampson N, Berger P (2008). Dysregulation of Dkk-3 expression in benign and malignant prostatic tissue. Prostate.

[30] Zenzmaier C, Sampson N, Plas E, Berger P (2013). Dickkopf-related protein 3 promotes pathogenic stromal remodeling in benign prostatic hyperplasia and prostate cancer. Prostate.

[31] Zenzmaier C, Heitz M, Klocker H, Buck M, Gardiner RA, Berger P (2011). Elevated levels of Dickkopf-related protein 3 in seminal plasma of prostate cancer patients. J Transl Med.

